# Spatial Heterogeneity Regulates Plant-Pollinator Networks across Multiple Landscape Scales

**DOI:** 10.1371/journal.pone.0123628

**Published:** 2015-04-09

**Authors:** Eduardo Freitas Moreira, Danilo Boscolo, Blandina Felipe Viana

**Affiliations:** 1 Zoology Department, Federal University of Bahia, UFBA, Salvador, Bahia, Brazil; 2 Faculty of Philosophy, Sciences and Literature of Ribeirão Preto, University of São Paulo, Ribeirão Preto, FFCLRP-USP, São Paulo, Brazil; University of New South Wales, AUSTRALIA

## Abstract

Mutualistic plant-pollinator interactions play a key role in biodiversity conservation and ecosystem functioning. In a community, the combination of these interactions can generate emergent properties, e.g., robustness and resilience to disturbances such as fluctuations in populations and extinctions. Given that these systems are hierarchical and complex, environmental changes must have multiple levels of influence. In addition, changes in habitat quality and in the landscape structure are important threats to plants, pollinators and their interactions. However, despite the importance of these phenomena for the understanding of biological systems, as well as for conservation and management strategies, few studies have empirically evaluated these effects at the network level. Therefore, the objective of this study was to investigate the influence of local conditions and landscape structure at multiple scales on the characteristics of plant-pollinator networks. This study was conducted in agri-natural lands in Chapada Diamantina, Bahia, Brazil. Pollinators were collected in 27 sampling units distributed orthogonally along a gradient of proportion of agriculture and landscape diversity. The Akaike information criterion was used to select models that best fit the metrics for network characteristics, comparing four hypotheses represented by a set of a priori candidate models with specific combinations of the proportion of agriculture, the average shape of the landscape elements, the diversity of the landscape and the structure of local vegetation. The results indicate that a reduction of habitat quality and landscape heterogeneity can cause species loss and decrease of networks nestedness. These structural changes can reduce robustness and resilience of plant-pollinator networks what compromises the reproductive success of plants, the maintenance of biodiversity and the pollination service stability. We also discuss the possible explanations for these relationships and the implications for landscape planning in agricultural areas.

## Introduction

Studies on biological interactions have helped us understand how the emergent properties of mutualistic networks can contribute to the stability of biological systems facing environmental variation [1]. However, despite the importance of the spatial-temporal distribution and behavior of interacting species as major determinants of network structure [2,3], only few studies have explicitly analyzed the direct effects of landscape structure on a key mutualistic inter-specific interaction network, such as a plant-pollinator network [1,4]. Consequently, we still have a poor understanding of the way in which the surrounding environmental conditions can interfere with this process at the systemic level [4–7].

Nevertheless, recent empirical evidence suggests that mutualistic networks may not react as robustly to changes in the landscape as previously expected [4,8]. Landscape modifications strongly affect cross-pollination and plant sexual reproduction primarily by reducing the diversity and availability of pollinators [4]. This mechanism is based on the increased spatial isolation of populations and decreased supplies of floral resources and nesting sites [6]. Furthermore, most agricultural crops on the planet can benefit from or are dependent on pollination services, implying that the negative effects of agriculture on pollinators also reduce agricultural productivity [5,7,9,10]. This negative feedback threatens the sustainability of long-term agricultural production, with the decrease of stability and growth of agricultural yield [11,12]. However, regardless of their important contributions, all these previous studies suffer from serious limitations in terms of establishing clear guidelines for land-use planning. These limitations result from several conceptual and methodological issues.

More specifically, there is a general lack of consensus on the negative effect of agriculture, given that results differ among biological groups, landscape definitions and study scales [1,4,6]. Most of these studies were based on a binary habitat-matrix landscape model, an approach which has strong limitations for many systems. Real landscapes are usually heterogeneous and much more complex than a representation with only two land cover classes. In addition, the organisms’ perception of their environment can vary greatly throughout their life history and among species. These principles imply that the definition of habitat must be species specific. Defining habitat for a whole community can thus be tricky and may not be feasible in many cases. A more realistic approach has been proposed by Fahrig et al [13], who suggest that the study of landscape effects on biological communities should be made from the perspective of landscape functional heterogeneity at proper scales. If we consider that communities are complex hierarchical systems, multi-level approaches become necessary for understanding the responses of the systems to disturbance [14]. Vegetation quality and landscape structure may affect the foraging behavior of pollinators at the local level [15,16], the number of pollinators present in the vicinity of the crops at a proximal surrounding landscape level [10,17] and the population’s dynamics at a broader landscape level, compatible with the maintenance of a large number of individuals [3,18]. Accordingly, the influence of each factor of interest at each level must be detected at its appropriate scale, and multiple-level approaches, which are uncommon in the literature, may be necessary to effectively understand these patterns [14,19].

In this study, we aimed to evaluate whether the characteristics of the plant-pollinator network are best explained by a relationship with the environmental characteristics at a single level or at multiple levels in a region with a diverse mosaic of agricultural and natural environments. More specifically, four alternative hypotheses were contrasted, three representing single-level influence (local vegetation, proximal landscape or broad landscape) and one representing multilevel influence. The four hypotheses were represented by corresponding models, and the best model was identified based on the Akaike information criterion through a model selection approach [20]. Additionally, to better understand the interplay between these factors, we performed exploratory analyses to evaluate the relationship between the conversion of natural vegetation into cultivated areas and the other features of the landscape. We then discuss the possible implications of these relationships for land use management and the development of guidelines to reconcile the productivity of agricultural systems with biological conservation, enabling more sustainable land occupation.

## Material and Methods

### Ethic issues and survey permits

We attest that the field studies did not involve endangered or protected species. In accordance with the environmental legislation of Brazil (norm n°. 154/2007) we received in 07/15/2011 an authorization (N° 12023–3, authentication code N° 16512688) from the Brazilian Federal Environmental Agency (Instituto Brasileiro do Meio Ambiente e dos Recursos Naturais Renováveis—IBAMA), for collection of biological material during the period between 10/2010 and 04/2012. For more details, please access the SISBIO (Sistema de Autorização e Informação em Biodiversidade), www.icmbio.gov.br/sisbio or contact the corresponding author (see above). In addition, the study was conducted on private land. For further information, please contact the Landowners Association Agropolo Mucuge/Ibicoara.

### Study area

**Fig 1:**
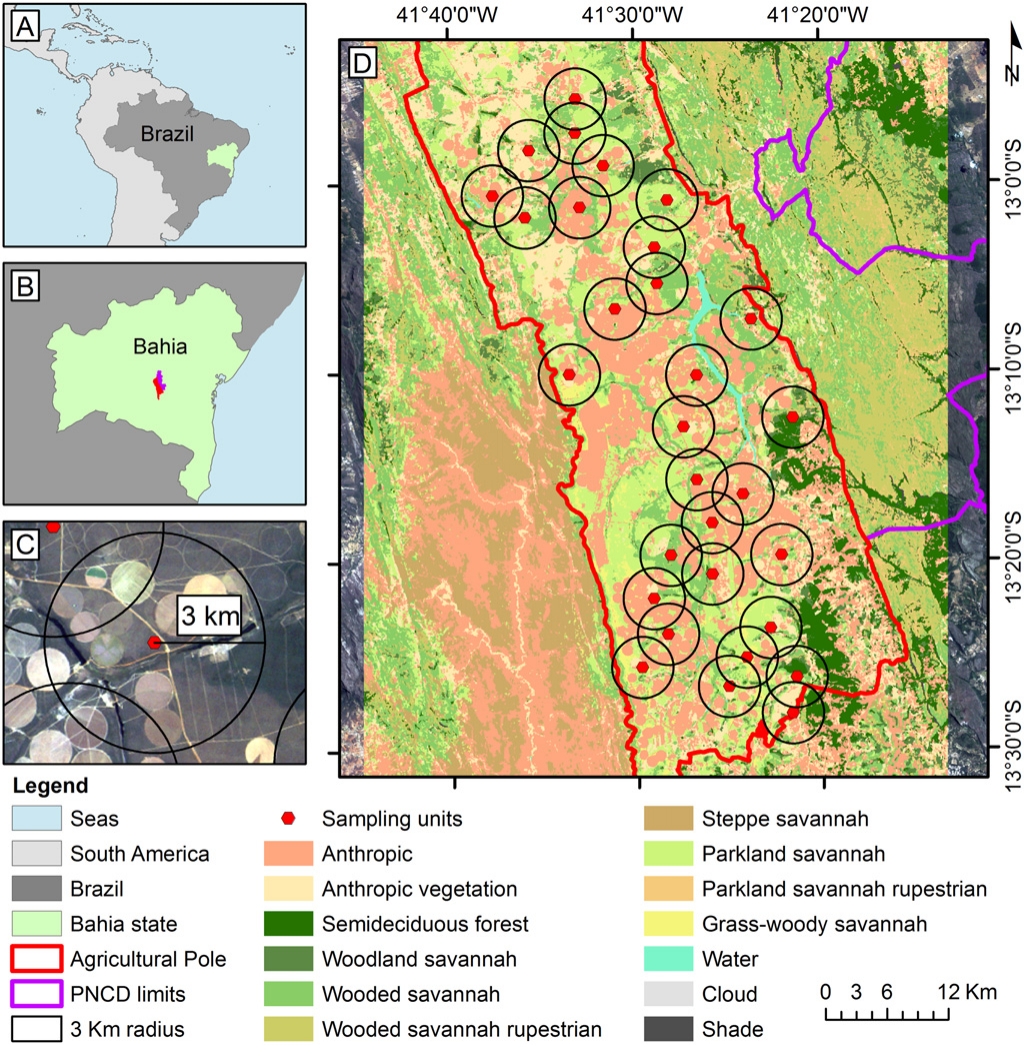
Left side: A—highlighted in green, dark gray and light gray, the state of Bahia, Brazil and South America, respectively; B—At the center of the state of Bahia (green), the studied region, with the lands of the agricultural partnership in red and Chapada Diamantina National Park in purple; C—example of the 3 km buffer used for the selection of sampling units; D—arrangement of the 27 selected sampling units (red dots) in the study region and the land cover classification used for the calculation of landscape metrics.

The present study was conducted on lands managed by the Landowners Association “Agropolo Mucuge/Ibicoara”, which is an agricultural partnership of farms in Chapada Diamantina, Bahia, Brazil occupying the flattest area (197,931 ha) of the municipalities of Ibicoara and Mucugê (limits: 41°42’11” W, 12°43’36” S; 41°15’5” W, 12°43’52” S; 41°42’51” W, 13°44’8” S; 41°15’40” W, 13°44’ 23” S; Fig 1). The altitude varies between 900 and 1400 m a.s.l. According to the Köppen-Geiger classification, the climate is tropical savannah (Aw). There are two well-marked seasons, and the rains are concentrated in the summer [21]. The area has an average annual precipitation of 1281 mm, an annual average maximum temperature of 29°C and a minimum temperature of 19.8°C [22]. This region is dominated by several types of Brazilian savannah (Cerrado), ranging from open meadows to semideciduous forests, with considerable floristic variation among the physiognomies and including parkland and wooded savannah, which are prevalent. Parkland savannah is characterized by graminoid formations interspersed with isolated nanophanerophytes, whereas the wooded savannah has a continuous hemicryptophyte layer with patches of sparse nanophanerophytes [23]. According to Raunkiaer’s nanophanerophytes are woody, dwarf and stunted plants, ranging from 0.25 of 5 m high, whereas hemicryptophytes are herbaceous plants with gametes and shoots protected at the soil level by leafs and sheaths which die out the dry season [23]. The native vegetation types occur in gradients and/or interspersed nearby patches forming mosaics with variable degrees of heterogeneity.

### Selection of sampling units

From a geographic information system (GIS) containing a SPOT image from the year 2008 (5 m spatial resolution) and information about the region's road network and field checks, 27 sampling units were selected (Fig 1, see also S1A Appendix). As criteria for this selection, we considered the density and stratification of the vegetation in each sampling unit and the proportion of cultivated area and landscape diversity, which was visually estimated from the image with a buffer of 3 km around each sampling point. The distribution of sampling units along the study area followed an orthogonal gradient between cultivated acreage and landscape diversity to minimize spatial autocorrelation. A buffer of 3 km was adopted as the minimum distance between sampling units (minimum nearest neighbor distance = 4.6 km, mean = 25.5 km, maximum = 63.6 km). The final distribution of sampling units tended toward dispersion (nearest neighbor ratio: 1.36, Z score: 3.75, p<0.001). These distances are consistent with the foraging range and dispersal distance of most Hymenoptera and are sufficient to minimize spatial pseudoreplication [10,17,24]. In addition, we sought a scattered distribution of sampling units, spatially alternating them with different values of the factors mentioned above and also avoiding bias due to differences in the age of landscape changes [25].

### Plant-pollinator surveys

For each sampling unit, a pair of collectors walked triangular routes within a reference hexagon divided into isosceles triangles with 25 m sides. The insects observed visiting flowers were collected along with samples of the visited plants for later identification. Sampling were conducted between 7:30 and 17:30 hours with entomological nets (for more detail, see also the S1B Appendix). During sampling, extra care was taken to record only visits in which anthers and/or stigmas were contacted. It is important to consider that, due to logistic constraints, no methods in addition to the training of the collectors were used to determine the nature of interactions, such as filming, photographic record, or specific protocols to determine pollinator efficiency. In addition, no information is available in the literature about the pollination biology of most native plants. However, Vázquez et al [26] have demonstrated that the frequency of interactions is often an effective measure of the pollination service provided, regardless of pollinator efficiency per visit.

For logistic reasons, the sampling units were divided into two groups homogeneously distributed throughout the study region. To avoid systematic effects of seasonality, each group was alternately sampled every two months between January and November 2011, twice in the rainy season and twice in the dry season. Ten hours per sample unit per campaign made a total of 40 h of sampling per unit. To avoid the possible effects of individual collectors, each collector gathered samples in different units during each field campaign.

### Network metrics

From the biological data, a frequency matrix was organized for each sampling unit. The network interaction indexes were calculated from this matrix. Three network indexes were selected based on their ecological relevance: number of interactions, weighted nestedness and Interaction strength asymmetry [1,27–29]. The total number of interactions was used because it is directly proportional to the level of ecological redundancy of the community and is related to the robustness and resilience of plant-pollinator networks [27,28,30]. Alternatively, the network-weighted nestedness index considers the unequal distribution of the interactions between the less and more connected species within the sides of the bipartite interaction network (plants or pollinators) [31]. A low-entropy network structure may increase the stability of the pollination system by anchoring most interactions on the most-connected pollinators and plants, which usually exhibit generalist behavior and are less susceptible to fluctuations [32,33]. Accordingly, we used an index based on overlap and decreasing fill to estimate the network weighted nestedness [31]. In contrast, the interaction strength asymmetry index reflects the dependency ratio among species of the different sides of the bipartite interaction network [28,34]. Although there is no consensus [27], interaction strength asymmetry can also enhance the stability of the pollination system by anchoring the most dependent species on the less dependent ones, thinning the risks associated with the dependence of the partner and acting in parallel with nestedness [28,32,34]. In addition, several authors have proposed that both types of asymmetries may be associated with network robustness and resilience [27,28,34]. Theoretically, a network’s robustness and resilience can be expressed in terms of the stability of the pollination system, with implications for its pollination efficiency [7,12,26].

Note that the high abundance of the invasive exotic species *Apis mellifera* can prevent the detection of landscape change effects on the native species’ network indexes. Studies have indicated that this bee can have a strong influence on the weighted network indexes [35]. Because the exotic *A*. *mellifera* exhibits highly generalist behavior, this bee tends to respond differently to environmental gradients than do native species [33,36]. This tendency may conceal the effect of population variations of native species on the network indexes most influenced by the frequency of visits. For this reason, the three network indexes were calculated considering the complete communities including the exotic species *A*. *mellifera* as well as the partial networks from which it was excluded. Finally, as part of an exploratory analysis, we evaluated the correlation between the number of links and the number of species (number of vertices) at each side of the interaction network and the number of links per species. All calculations were performed in R version 2.15.0, ‘bipartite’ package version 1.17 [37].

### Environmental multilevel quantification

To identify the most likely hypothesis about the influence of environmental characteristics on plant-pollinator networks, it was necessary to quantify the descriptive variables for three levels of influence (local vegetation, proximal landscape and broad landscape). It was also necessary to select the best scale of estimation for each level of influence from an interval of measurement scales that reflected the spatial variation of the ecological processes of interest [14,19]. For example, given that most foraging Hymenoptera experience the environment at a scale of a few tens of meters, it was estimated that the adequate scale for estimating the local vegetation was between 25 and 150 m [15].

Following the same logic, to estimate the landscape structure at the proximal level, we used scales ranging from 250 to 3,000 m, and to estimate the landscape at the broad level, we used scales ranging from 6,000 to 12,000 m [3,10,17,19,24]. The scales of the landscape at the proximal level (from 250 to 3,000 m) are compatible with the home range of most Hymenoptera, and it is expected that the landscape structure at this level will affect the possibility or the cost of residence of individuals of different species [10,17,24]. Finally, the scales of the landscape at the broad level (from 6,000 to 12,000 m) comprise areas large enough to hold a sufficient number of individuals to influence their population dynamics and distribution [14,19]. At this level, the landscape could promote concentration or dispersal of populations, as well as relatively high beta diversity, which can reduce the effect of local factors on the structure of communities if compared with low-beta-diversity regions [3].

#### Local vegetation conditions

To evaluate the effects of local vegetation (LV) on the characteristics of plant-pollinator networks, two principal aspects were considered: plant richness and vegetation structure [15,16]. A remote sensing technique was adopted to measure plant richness, vegetation structure and productivity using the two-band enhanced vegetation index (EVI2). This index was calculated from the physical reflectance values of bands corresponding to the red and near-infrared wavelengths from LANDSAT 5 satellite images taken on 06/02/2001 (wet season), which were atmospherically corrected and geometrically and radiometrically calibrated [38]. The calculation of the mean vegetation index (MEVI) was conducted at multiple scales, with circular buffers ranging between 25 and 150 m with a 25 m progression from the center of the sampling unit. From correlations with field-collected data (N = 11), it was possible to verify that the index EVI2 was a good surrogate measure of the number of branches (df = 9, r = 0.79, P<0.005) and plant richness (df = 9, r = 0.72, P = 0.01) for savannah physiognomies in the studied region.

#### Landscape structure at proximal and broad levels

To assess the influence of landscape structure on the characteristics of plant-flower-visitor networks, a land use map was produced from the supervised classification of LANDSAT 5 images dated 14/09/2011 (same year as the biological data collection). In total, 13 classes were used, including nine classes of vegetation. The vegetation classes were established following the system proposed by Veloso *et al* [23] for the classification of Brazilian vegetation, with certain classes requiring modification for the present study. The classes are as follows: anthropic vegetation (abandoned areas recently occupied by ruderal vegetation), grass-woody savannah, parkland savannah, wooded savannah, woodland savannah, semideciduous forest, parkland savannah on rock surface (rupestrian), wooded savannah on rock surface (rupestrian), steppe savannah, anthropic use (mainly agriculture but also including roads, buildings and anthropogenic bare soil), water and clouds and shades (Fig 1). Field data and pattern recognition of different targets that are distinguishable in the images were used for pixel sampling and training for the classification algorithm. For these procedures, we used the software ArcGIS 9.3 ESRI 2008 and the Maximum Likelihood Classification algorithm [39], which is available in the software ENVI 4.7 ITT 2009.

Once the land-use map was obtained, three metrics describing landscape structure were calculated. To represent landscape composition, we chose two indexes. the proportion of the area covered by the class Anthropic was used to describe the landscape proportion of agricultural area (PA) as a surrogate for the loss of native vegetation. The landscape Shannon’s diversity index was used to describe the landscape diversity (LD) because of its sensitivity to the presence of relatively rare landscape units, such as riparian environments and small dense shrub patches, which may be important for floral visitors. In addition, the area-weighted mean shape of landscape patches was used to describe the landscape configuration (LC) [40]. Landscape metrics were calculated using the module Patch Analyst Queens Press, Ontario Ministry of Natural Resources, 2012 in ArcGIS 9.3 ESRI 2008.

#### Detecting adequate spatial scales

As explained above, each of the three levels of influence comprises a range of scales that may be associated with a given ecological process. To select the most appropriate spatial scale for each of the three levels of influence, the best explanatory power (R^2^) was adopted as the criterion. To detect the best explanatory measurement scale for the combinations of factors and network characteristics, we subjected the results of the different radii of MEVI and surrounding landscape metrics in the two levels to a bootstrapping procedure, which consisted of selecting random subsets of 20 sampling units out of the original group of 27 to extract the value of R^2^ from simple linear regressions, using the environmental factors as explanatory variables and network metrics as response variables [20]. These procedures were repeated 1,000 times, generating distributions of R^2^ values that allowed us to select the scale with the highest mean R^2^ for each variable combination (see S1 Dataset and S1 Table).

### Multiple-level effects on networks

To compare the four alternative hypotheses for the relationship between the surrounding environmental conditions with characteristics of the plant-pollinator networks, a model selection technique using the Akaike information criterion was adopted based on information theory and maximum likelihood estimates [20]. For this, a specific group of mathematical models represented each hypothesis (Table 1). The first hypothesis states that the networks are regulated by the characteristics of the local vegetation, and is associated with the model group G1 composed by only one model, since vegetation characteristics are represented through a single synthetic variable. The second hypothesis, which states that the characteristics of networks are regulated by the landscape structure at the proximal level, was represented by the model group G2 with six models, which consisted of combinations of the three landscape indexes measured in the vicinity of the sampling points. The third hypothesis is that the characteristics of networks are regulated by the landscape structure at the broad level and was represented by the group G3, also composed by six models, with combinations of the three characteristics of the landscape at larger buffers. The fourth hypothesis states that the characteristics of networks are defined by a combination of factors at different levels that act on different traits of organisms, was represented by the group G4, with 24 models as a result of combinations of factors of different levels, with the restriction that each model could have only one factor at each level and that the models should encompass at least two levels. Finally, a null model without variables and with only a constant parameter was used to represent the lack of effects in order to determine whether the evaluated models were actually better than random variations [20]. The set of representative models plus the null model totals 40 (Table 1).

**Table 1:** List of models associated with their respective hypotheses.

Model group	Model	Parameters
**G1**	**Local vegetation**	
	***y = β***_***0***_ ***+ β***_***1***_ ***LV***	3
**G2**	**Proximal landscape structure**	
	***y = β***_***0***_ ***+ β***_***1***_ ***PPA + β***_***2***_ ***PLC + β***_***3***_ ***PLD***	5
	***y = β***_***0***_ ***+ β***_***1***_ ***PPA + β***_***2***_ ***PLC***	4
	***y = β***_***0***_ ***+ β***_***1***_ ***PPA + β***_***2***_ ***PLD***	4
	***y = β***_***0***_ ***+ β***_***1***_ ***PLC + β***_***2***_ ***PLD***	4
	***y = β***_***0***_ ***+ β***_***1***_ ***PPA***	3
	***y = β***_***0***_ ***+ β***_***1***_ ***PLC***	3
	***y = β***_***0***_ ***+ β***_***1***_ ***PLD***	3
**G3**	**Broad landscape structure**	
	***y = β***_***0***_ ***+ β***_***1***_ ***BPA + β***_***2***_ ***BLC + β***_***3***_ ***BLD***	5
	***y = β***_***0***_ ***+ β***_***1***_ ***BPA + β***_***2***_ ***BLC***	4
	***y = β***_***0***_ ***+ β***_***1***_ ***BPA + β***_***2***_ ***BLD***	4
	***y = β***_***0***_ ***+ β***_***1***_ ***BLC + β***_***2***_ ***BLD***	4
	***y = β***_***0***_ ***+ β***_***1***_ ***BPA***	3
	***y = β***_***0***_ ***+ β***_***1***_ ***BLC***	3
	***y = β***_***0***_ ***+ β***_***1***_ ***BLD***	3
**G4**	**Multi-level combined effect**	
	***y = β***_***0***_ ***+ β***_***1***_ ***LV + β***_***2***_ ***PPA + β***_***3***_ ***BPA***	5
	***y = β***_***0***_ ***+ β***_***1***_ ***LV + β***_***2***_ ***PPA + β***_***3***_ ***BLC***	5
	***y = β***_***0***_ ***+ β***_***1***_ ***LV + β***_***2***_ ***PPA + β***_***3***_ ***BLD***	5
	***y = β***_***0***_ ***+ β***_***1***_ ***LV + β***_***2***_ ***PLC + β***_***3***_ ***BPA***	5
	***y = β***_***0***_ ***+ β***_***1***_ ***LV + β***_***2***_ ***PLC + β***_***3***_ ***BLC***	5
	***y = β***_***0***_ ***+ β***_***1***_ ***LV + β***_***2***_ ***PLC + β***_***3***_ ***BLD***	5
	***y = β***_***0***_ ***+ β***_***1***_ ***LV + β***_***2***_ ***PLD + β***_***3***_ ***BPA***	5
	***y = β***_***0***_ ***+ β***_***1***_ ***LV + β***_***2***_ ***PLD + β***_***3***_ ***BLC***	5
	***y = β***_***0***_ ***+ β***_***1***_ ***LV + β***_***2***_ ***PLD + β***_***3***_ ***BLD***	5
	***y = β***_***0***_ ***+ β***_***1***_ ***LV + β***_***2***_ ***PPA***	4
	***y = β***_***0***_ ***+ β***_***1***_ ***LV + β***_***2***_ ***PLC***	4
	***y = β***_***0***_ ***+ β***_***1***_ ***LV + β***_***2***_ ***PLD***	4
	***y = β***_***0***_ ***+ β***_***1***_ ***LV + β***_***2***_ ***BPA***	4
	***y = β***_***0***_ ***+ β***_***1***_ ***LV + β***_***2***_ ***BLC***	4
	***y = β***_***0***_ ***+ β***_***1***_ ***LV + β***_***2***_ ***BLD***	4
	***y = β***_***0***_ ***+ β***_***1***_ ***PPA + β***_***2***_ ***BPA***	4
	***y = β***_***0***_ ***+ β***_***1***_ ***PPA + β***_***2***_ ***BLC***	4
	***y = β***_***0***_ ***+ β***_***1***_ ***PPA + β***_***2***_ ***BLD***	4
	***y = β***_***0***_ ***+ β***_***1***_ ***PLC + β***_***2***_ ***BPA***	4
	***y = β***_***0***_ ***+ β***_***1***_ ***PLC + β***_***2***_ ***BLC***	4
	***y = β***_***0***_ ***+ β***_***1***_ ***PLC + β***_***2***_ ***BLD***	4
	***y = β***_***0***_ ***+ β***_***1***_ ***PLD + β***_***2***_ ***BPA***	4
	***y = β***_***0***_ ***+ β***_***1***_ ***PLD + β***_***2***_ ***BLC***	4
	***y = β***_***0***_ ***+ β***_***1***_ ***PLD + β***_***2***_ ***BLD***	4
**Null model**	**No effect**	
	***y = β***_***0***_	2

G1—Local vegetation; G2—Proximal landscape structure; G3—Broad landscape structure; G4 Multi-level combined effect; Null model—no effect; *β*_*0*_—intercept; *β*_*1*_, *β*_*2*_ and *β*_*3*_—parameters associated with the respective variables; *LV*—local vegetation; *PPA*—Proximal landscape proportion of agricultural cover; *PLC*—Proximal landscape configuration; *PLD*—Proximal landscape diversity; *BPA*—Broad landscape proportion of agricultural cover; *BLC*—Broad landscape configuration; *BLD*—Broad landscape diversity; The number of parameters presented at the table are for the normal distribution used for interaction strength asymmetry and nestedness analysis; considering that the Poisson’s distribution was used for the number of links, in this case it is necessary to subtract one from the number of parameters for each model.

To reduce the influence that the range of variation in the absolute values of the factors might have on the estimation of model parameters, each independent variable was equally scaled and centered to zero by subtracting its mean value and then dividing it by its standard deviation. This transformation preserves the original characteristics of the variables while adjusting to comparable intervals (S1 Dataset). The maximum likelihood functions were calculated from generalized linear models (GLMs) because these models allow flexible choices of error distributions. We adopted the Poisson distribution for the network’s number of interactions because it can be applied to count data. Gaussian distributions were adopted for the other continuous dependent variables [41].

The models were compared using the values of the second-order Akaike information criterion (AICc), which is suitable for small samples (n <40). The delta AICc (Δi) value for each model, namely, the difference between the AICc value for that model and the lowest AICc value in the set of models, was used to evaluate the plausibility of the candidate models. Models with values of delta AICc (Δi) <2 were considered equally plausible. We also considered the Akaike weights (Wi) of the models, the evidence ratio in relation to the minimum AICc model (W1/Wj) and the importance of the variables in each selection process (ΣW) to evaluate the normalized differences between the equally plausible models as well as the differences relative to the null model [20]. The model selection analyses were performed in R version 2.15.0, package ‘bbmle’ version 1.0.16 [20].

## Results

We collected 2570 individuals of pollinators belonging to 216 species of Hymenoptera. These pollinators visited 115 plant species belonging to 47 families. The most abundant flower visitors were *Apis mellifera* Linnaeus (1758), representing 46.4% of the total sample; *Trigona spinipes* (Fabricius, 1793), representing 9.4%; *Vespidae sp1*, representing 4.2%; and *Geotrigona sp1*, representing 2.1%. *Apis mellifera* was the only species collected in all 27 sampling units and was the most abundant in 21 units. The most visited plants were *Eriope salviifolia* (Pohl ex Benth) Harley (Lamiaceae), with 364 visits (13.5%); *Tachigali paniculata* Aubl (Fabaceae), with 206 visits (7.6%); *Baccharis retusa* DC. (Asteraceae), with 199 visits (7.4%); *Eremanthus capitatus* (Asteraceae), with 167 visits (6.2%); *Croton campestris* (Euphorbiaceae), with 161 visits (6%); and *Allagoptera campestris* (Mart.) Kuntze (Arecaceae), with 150 visits (5.5%). The most widely distributed species were *Hyptis crassifolia* Mart. (Lamiaceae), which occurred in 17 sampling units, and *Borreria verticillata* (L.) G. Mey (Rubiaceae), which occurred in 15 sampling units.

The total number of species in the networks ranged from 16 to 56. The network with the lowest pollinator richness had six species and that with the highest had 40. The number of plant species ranged from seven to 21 in these networks. The number of interactions ranged between 16 and 77 edges, and the average number of edges per species ranged from 0.76 to 1.25. Sixty-five per cent of the variation in the number of edges (R^2^ = 0.65; p<0.001) could be explained by the total number of species, and 67% could be explained by the number of pollinator species (R^2^ = 0.67; p<0.001). However, there was no clear correlation between the number of interactions and the number of plant species (R^2^ = 0.17; p = 0.03), indicating that the main factor responsible for the increase in the number of interactions was the addition of floral visitors to the networks. The mean number of links per species was positively correlated with the number of interactions, which explained 40% of its variation (R^2^ = 40; p <0.001). Although the interaction strength asymmetry showed values ranging from -0.44 to 0.41, most networks presented positive values, indicating a stronger dependency of pollinators relative to plants than plants relative to pollinators. Only 10 networks showed negative values of asymmetric dependence.

### Response of plant-pollinator networks to environmental effects at multiple levels

As expected, the general patterns are clearer when the invasive exotic species *A*. *mellifera* was excluded from the networks (Table 2; see also S2 Appendix and S2 to S7 Tables). Therefore, we decided to describe and discuss in more detail the results containing only the native pollinator’s interactions. For all three networks characteristics analyzed, the models with the lower AICc were from the group G4. This results supports the fourth hypothesis which states that the network characteristics are defined by a combination of factors at different levels that act on different biological aspects of the organisms (Table 2, S3 Table, S5 Table and S7 Table).

**Table 2:** Summary of model selection for each dependent variable, showing the models with ΔAICc <2 and the subsequent model.

Network metric	Model group	Model	ΔAICc	AICcWi	Wi/Wk
**Number of interactions complete**	G4	***y = β***_***0***_ ***+ β***_***1***_ ***LV + β***_***2***_ ***PLD***	0	0.26	35.3
	G2	***y = β***_***0***_ ***+ β***_***1***_ ***PLD***	1.4	0.13	17.6
	G4	***y = β***_***0***_ ***+ β***_***1***_ ***LV + β***_***2***_ ***PLD + β***_***3***_ ***BLC***	2.9	0.06	8.3
**Number of interactions without *A*. *mellifera***	G4	***y = β***_***0***_ ***+ β***_***1***_ ***LV + β***_***2***_ ***PLD***	0	0.23	75.2
	G4	***y = β***_***0***_ ***+ β***_***1***_ ***PLD + β***_***2***_ ***BLC***	0.9	0.15	47.7
	G2	***y = β***_***0***_ ***+ β***_***1***_ ***PLD***	1.2	0.13	41.1
	G4	***y = β***_***0***_ ***+ β***_***1***_ ***LV + β***_***2***_ ***PLD + β***_***3***_ ***BLC***	1.7	0.1	31.4
	G4	***y = β***_***0***_ ***+ β***_***1***_ ***LV + β***_***2***_ ***PLD + β***_***3***_ ***BLD***	2	0.08	27.2
**Nestedness complete**	G4	***y = β***_***0***_ ***+ β***_***1***_ ***PLD + β***_***2***_ ***BLD***	0	0.12	1.7
	G3	***y = β***_***0***_ ***+ β***_***1***_ ***BPA + β***_***2***_ ***BLD***	0.3	0.11	1.5
	G3	***y = β***_***0***_ ***+ β***_***1***_ ***BLD***	0.7	0.09	1.2
	Null model	***y = β***_***0***_	1.1	0.07	1
	G3	***y = β***_***0***_ ***+ β***_***1***_ ***BLC + β***_***2***_ ***BLD***	1.1	0.07	-
	G3	***y = β***_***0***_ ***+ β***_***1***_ ***BPA + β***_***2***_ ***BLC + β***_***3***_ ***BLD***	1.7	0.05	-
	G2	***y = β***_***0***_ ***+ β***_***1***_ ***PLD***	2.6	0.03	-
**Nestedness without *A*. *mellifera***	G4	***y = β***_***0***_ ***+ β***_***1***_ ***PLC + β***_***2***_ ***BLD***	0	0.47	46.8
	G4	***y = β***_***0***_ ***+ β***_***1***_ ***LV + β***_***2***_ ***PLC + β***_***3***_ ***BLD***	3	0.1	10.2
**Network strength asymmetry complete**	G2	***y = β***_***0***_ ***+ β***_***1***_ ***PLC***	0	0.14	1.9
	Null model	***y = β***_***0***_	1.3	0.07	1
	G4	***y = β***_***0***_ ***+ β***_***1***_ ***LV + β***_***2***_ ***PLC***	1.8	0.06	0.8
	G3	***y = β***_***0***_ ***+ β***_***1***_ ***BPA***	2	0.05	0.7
**Network strength asymmetry without *A*. *mellifera***	G4	***y = β***_***0***_ ***+ β***_***1***_ ***PLD + β***_***2***_ ***BLD***	0	0.35	22.7
	G3	***y = β***_***0***_ ***+ β***_***1***_ ***BLD***	2	0.13	8.3

ΔAICc—differences in AICc relative to the lowest value of AICc of all models; AICcW_i_—Akaike weight of model i; W_i_ / W_k_—ratio between the weight of model i and the weight of the null model K; G1—local vegetation; G2—proximal landscape; G3—Broad landscape; G4—Combined effect; ***β***_***0***_—intercept; ***β***_***1***_, ***β***_***2***_ and ***β***_***3***_—parameters associated with the respective variables; ***LV***—local vegetation; ***PPA***—Proximal landscape proportion of agricultural cover; ***PLC***—Proximal landscape configuration; ***PLD***—Proximal landscape diversity; ***BPA***—Broad landscape proportion of agricultural cover; ***BLC***—Broad landscape configuration; ***BLD***—Broad landscape diversity.

#### Number of interactions

**Fig 2:**
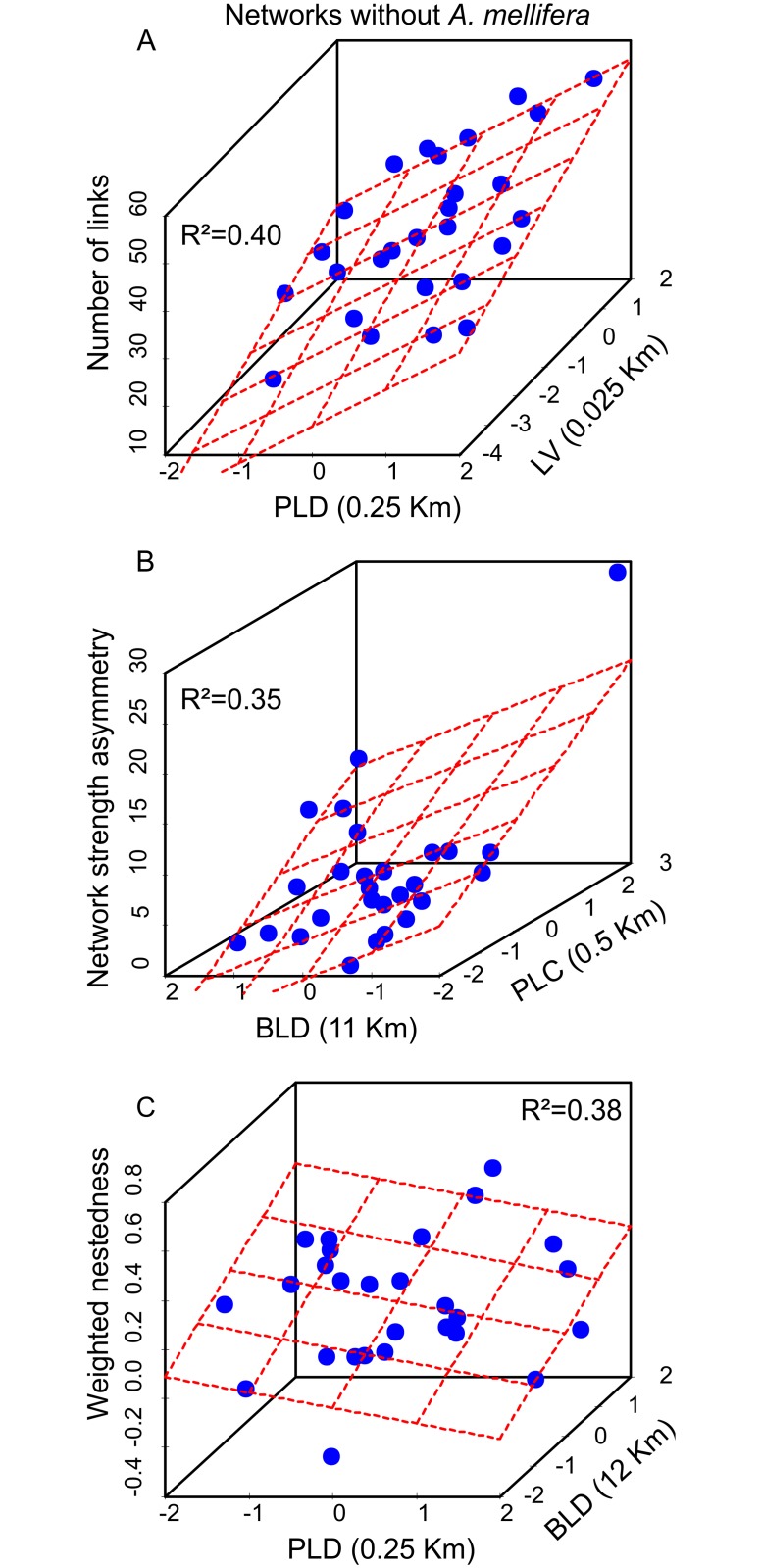
Relationship between the characteristics of networks (Y axis) and selected models (X axis): A—Number of links of networks; B—Network interaction strength asymmetry; C—network nestedness; LV—Local vegetation; PLD—Proximal landscape diversity; PLC—Proximal Landscape configuration; BLD—Broad landscape diversity

The number of interactions was positively influenced by the local vegetation structure and landscape diversity at the proximal level as shown by the best model (Fig 2A, see also S3 Table). This best model was 100% more plausible and 28% more explanatory than landscape diversity alone, which was the second in the rank of equally plausible models (AICcΔi <2; Tables 2 and 3). Note that the second and fourth equally plausible models included the additive positive effect of landscape configuration at the broad level in addition to the variables mentioned above but had no additional explanatory power compared to the best model (R^2^ = 0.43 and R^2^ = 0.40 respectively).

**Table 3:** Importance value (Σwi) of each independent variables for each model selection group.

Network metric	LV	PLD	PLC	PPA	BLD	BLC	BPA
Number of interactions complete	0.57*	0.74**	0.31	0.21	0.2	0.21	0.17
Number of interactions without *A*. *mellifera*	0.49*	0.88**	0.17	0.23	0.27	0.44*	0.23
Nestedness complete	0.22	0.33	0.19	0.29	0.79**	0.29	0.33
Nestedness without *A*. *mellifera*	0.29	0.13	0.71*	0.38	0.83**	0.13	0.24
Network strength asymmetry complete	0.37	0.31	0.38	0.25	0.3	0.15	0.23
Network strength asymmetry without *A*. *mellifera*	0.17	0.66**	0.2	0.17	0.82**	0.24	0.23

**Variables that were part of the models with ΔAICc <2 with ∑*w*_*i*_<0.60;

*variables that were part of the models with ΔAICc <2 with ∑*w*_*i*_<0.40; LV—local vegetation; PPA—Proximal landscape proportion of agricultural cover; PLC—Proximal landscape configuration; PLD—Proximal landscape diversity; BPA—Broad landscape proportion of agricultural cover; BLC—Broad landscape configuration; BLD—Broad landscape diversity.

#### Nestedness

The nestedness was influenced by landscape heterogeneity at both the proximal and broad levels (Table 2, Fig 2B). The best model included the positive effect of landscape configuration heterogeneity at the proximal level and the negative effect of landscape diversity at the broad level (AICcΔi <2, AICcWi = 0.47, Table 2; see also S5 Table). Furthermore, these two variables had higher values of importance in the model selection process compared with the other variables (Table 3).

#### Network interaction strength asymmetry

The network interaction strength asymmetry presented a pattern opposite to that seen for nestedness (Fig 2C). It was strongly negatively influenced by landscape diversity at the proximal level and positively influenced by landscape diversity at the broad level, as shown by the best model (Table 2; see also S7 Table). These two variables were also the most important compared with the other variables (Table 3).

#### The importance of agriculture

The proportion of agriculture was not a major factor for any of the analyzed networks (Table 3). Nevertheless, our data suggest that the conversion of native vegetation in agricultural areas may have an important role in changing the configuration and composition of landscapes, which indirectly affects the interaction networks. Exploratory analyses showed that intermediate conversion levels of natural environment into crop areas could cause increased landscape heterogeneity and diversity in certain cases (S3 Appendix). However, this association is only present at the proximal landscape level and for proportion of agriculture roughly lower than approximately 40%. Above this value, the relationship tend to be inverse, with landscape heterogeneity decreasing with increases in the proportion of agriculture in the landscape (see more details in S3 Appendix).

## Discussion

The association of local vegetation and landscape heterogeneity at multiple levels strongly influenced the structure of the plant-pollinator networks we studied. Each analyzed network characteristic was influenced and best explained by a combination of different factors at different levels that may be related to specific biological processes [14,42]. It is already known that multiple-level explanations are essential to the understanding of the functioning and regulation of complex systems, such as biological communities [14,42], but the interplay of factors at multiple levels has been largely overlooked [19,43]. Here, we present possible explanations for the observed patterns according to foraging [15,16] and habitat heterogeneity theories [44], as well as to the latest proposals on landscape heterogeneity [13,45].

**Fig 3:**
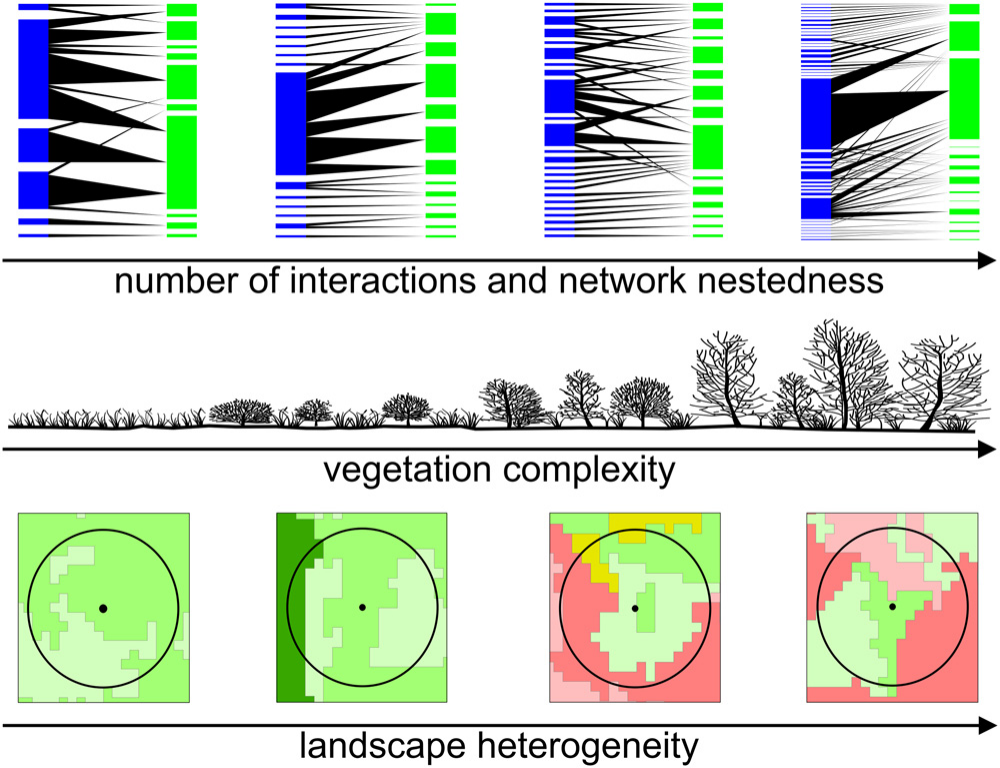
Scheme showing the qualitative relationship between landscape heterogeneity, vegetation heterogeneity and the networks structure.

According to our results, plant-pollinator networks tend to be larger, more connected and more nested as environmental heterogeneity increases (Fig 3). Because pollinators can memorize their surrounding environment and properly respond to it, when local plant richness, density and productivity increase at the local level, pollinators residence time will also be longer [15,16]. In association with the amount of plants, long residence time will raise the probability of plant-pollinator encounters [2,26,46], what can explain the positive relation of local vegetation heterogeneity with the number of interactions. At another level, the positive effect of proximal landscape diversity is most likely associated with the maintenance of pollinators nesting in the surroundings of the resource patches. Discontinuous availability of nearby nesting and floral resources may be a limiting factor for many species of flower visitors [24,47]. Limitation can arise from synchronized vegetation phenology in different environment types. However, if plant phenology is asynchronous, there will be variation in the spatial and temporal availability of these resources [48]. In this situation, when crucial resources are absent or scarce in certain types of environments, they may be replaced by alternative resources available at other nearby place [13,45]. As a consequence, more heterogeneous landscapes favor the maintenance of a greater number of species with different requirements [13,45].

The patterns found for nestedness and interaction strength asymmetry appear to be entangled and may reflect compensation for the increase in competitive pressure given the increase in pollinator species richness promoted by the flux of organisms between the landscape units. The positive effect of more heterogeneous landscape configuration on network nestedness at the proximal level can be explained from pollinators foraging patterns. Landscapes with more irregular edges and higher interspersion of elements tend to have smaller distances between any two points belonging to different vegetation or land use types. As a result, these landscapes can promote easier flux of individuals. However, these individuals will tend to visit mainly the plants that produce massive flowering [15,16]. In our study, these plants were actually the most connected, contributing to an increase in network nestedness [35].

Interestingly, given that a wider range of visitors was present to use these resources, possible the niche overlap would increase the pressure of competition [36,49]. If there is high competitive pressure in heterogeneous landscapes, the niches of the most generalist species are expected to change [50]. Some individuals of these species may tend to expand their diets to include less attractive plants, offsetting the niche overlap with other species [50]. As generalist species tend to be more abundant, they place a greater weight on the network asymmetry than the less abundant species [35,51]. These mechanisms may explain how an increase in landscape heterogeneity could promote an increase in network size, number of connections and nestedness while simultaneously decreasing the interaction strength of asymmetry.

At the broad landscape level, we found that the influence of heterogeneity was opposite to that found at the proximal level, suggesting the existence of a buffering effect [18]. While complex landscapes at the proximal level favor the concentration of larger populations in a certain location, landscape heterogeneity at the broad level can promote dilution of flower visitors in a very large area, minimizing the landscape effect at the proximal level [3].

The increase in biological diversity promoted by the flow of individuals between different landscape units in these heterogeneous landscapes may lead to higher intensity of ecological processes and the reduction of their temporal variability. The possible existence of hierarchical buffering effects among landscape levels are in accordance with the "spatial insurance" hypothesis [18]. In this case, biodiversity shall acts as an "insurance" for ecosystem functions through functional redundancy, buffering the environmental variations with functional compensation. This phenomenon may be caused entirely by spatial dynamics in heterogeneous landscapes, as suggested by metacommunity modelling studies [18].

Moreover, based on our results, we can theorize beyond the role of functional redundancy and incorporate other structural characteristics of the plant-pollinator networks in the framework of the “spatial insurance” hypothesis. For example, nestedness and interaction strength asymmetry can also enhance the stability of plant-pollinator networks by increasing the robustness and resilience of the system [27,30,32]. Therefore, the spatial insurance promoted by multi-level heterogeneity can be even more important than previously conceived [52].

### Consequences for management and landscape planning

Because flower visitors are responsible for the pollination of many crops, the patterns found in this study have important implications for the conservation of pollination services and can contribute to landscape design directives, which may directly affect the productivity of most agricultural crops on the planet [5,7,9,10]. The “spatial insurance” promoted by multi-level landscape heterogeneity, as described in the previous section, can have a critical effect on crop productivity once it influences pollination service stability [12].

We found evidence that in landscapes (≈20 hectares) with more than 40% of the total area devoted to agriculture there was a tendency toward environmental homogenization. This tendency may have an indirect negative effect on the maintenance of many flower visitor groups, especially for certain groups of social and solitary bees that nest in pre-existing cavities [52]. This is in accordance with the recently suggested thresholds for pollinators and interactions debt in plant-pollinator networks due to habitat loss from 40% to 60% [30,53]. In this context, the isolation of crops may affect the stability of pollination services [3,7,9,10,12,33,54]. Therefore farmers must continuously increase acreage to maintain the yield of these crops, creating a negative feedback loop [12] leading to increased monetary and environmental production costs.

However, we can also conclude that, at a proximal landscape level, intermediate proportions (between 0 and 40%) of anthropic environments may be associated with increased landscape diversity, indirectly favoring the maintenance of a greater diversity of floral visitors and an increased number of interactions in plant-pollinator networks. Other studies have found similar evidence, with cultures in full bloom and fallow strips representing important sources of food and nesting sites for certain groups of pollinators but not for all [54,55].

This landscape context-dependent effect of agricultural areas indicates that it is possible to plan agricultural landscapes that can balance the trade-offs between production area and productivity. In addition, landscape heterogeneity at broader landscape levels (thousands of hectares) can buffer the influence of local structure and surrounding environments. This implies that when planning agricultural landscapes, decision makers must aim to increase the heterogeneity of landscape composition and configuration, considering the spatial arrangement of landscape elements as well as the multiple levels of interference [56]. The creation of general policies to regulate land use accordingly to that would be beneficial to agroecosystem depending on pollination services. In Brazil, for instance, legislation demands that in the Cerrado domain at least 35% of each rural estate area must be preserved as natural environment (20% within the estate and other 15% within the same watershed). Even though with current the lacks of concern regarding the landscape heterogeneity and the underestimation of land cover percentages, this kind of law brings some advances. If generally applied with the necessary regional adjustments, such measures could bring mutual benefits to farmers and for biodiversity maintenance worldwide.

## Supporting Information

S1 Appendix Details of sampling unit selection and sampling methods. (DOC)

S2 Appendix Details of the comparison between the complete plant-pollinator networks and after the exclusion of *Apis mellifera* Linnaeus (1758) (DOC)

S3 Appendix Relationship between the proportion of agriculture and the landscape structure. (DOC)

S1 Dataset Datasets for model selection. (XLS)

S1 Table Scale selection results. (XLS)

S2 Table Model selection ranking for number of interactions with the complete networks. (DOC)

S3 Table Model selection ranking for number of interactions without the species *Apis mellifera* Linnaeus (1758). (DOC)

S4 Table Model selection ranking for network nestedness with the complete networks. (DOC)

S5 Table Table. Model selection ranking for network nestedness without the species *Apis mellifera* Linnaeus (1758). (DOC)

S6 Table Model selection ranking for network interaction strength asymmetry with the complete networks. (DOC)

S7 Table Model selection ranking for network interaction strength asymmetry without the species *Apis mellifera* Linnaeus (1758). (DOC)
