# Supplementary material for: Spatial Heterogeneity Regulates Plant-Pollinator Networks across Multiple Landscape Scales
Source: PLoS One. 2015 Apr 9;10(4):e0123628. doi: 10.1371/journal.pone.0123628 (PMC4391788; doi:10.1371/journal.pone.0123628)
Supplement: S1 Appendix — (DOC) [file pone.0123628.s001.doc]

**Spatial heterogeneity regulates plant-pollinator networks across multiple landscape scales**

Eduardo Freitas Moreira^1^*, Danilo Boscolo^2^, Blandina Felipe Viana^1^

1 Zoology Department, Federal University of Bahia, UFBA, Salvador, Bahia, Brazil

2 Faculty of Philosophy, Sciences and Literature of Ribeirão Preto, University of São Paulo, Ribeirão Preto, FFCLRP-USP São Paulo, Brazil

* eduardofreitasmoreira@gmail.com

**Appendix S1 – Details of sampling unit selection and sampling methods**

**A - Sampling unit selection**

A Geographic Information System (GIS) and field verifications were used to select 27 sampling units (Figure S1). To certify that the variation of landscape structure around our sampling points would follow our desired study design, an orthogonal gradient of proportion of cultivated area and landscape diversity was sought within circles with three km radius centered at each sampling unit. For each circle a visual estimate of these landscape features from a color composite map from SPOT satellite images (2008) with high spatial resolution (5 m pixels) was used (Figure S1). To avoid spatial pseudoreplication, we sought to intersperse the values of previously cited factors (1). Also, to avoid spatial autocorrelation, we adopted three km as the minimum distance between the sampling units (2–5). The pre-selected sampling units in the GIS were evaluated in the field and, if vegetation type in these points were qualitatively different from expected, the point was displaced tens to hundreds of meters to the nearest location with similar desired characteristics.


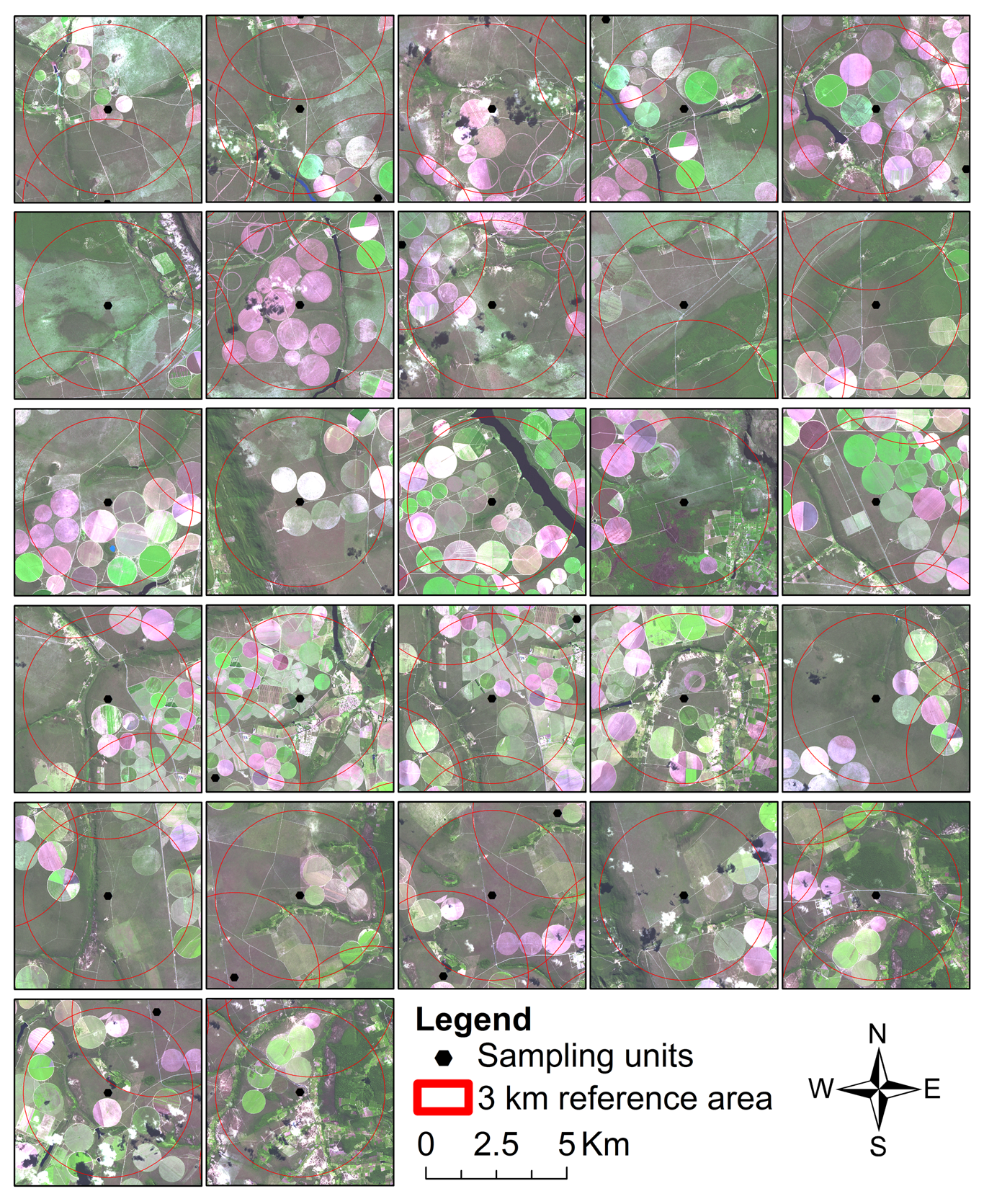
Figure S1 - The 27 sampling units selected, representing the orthogonal gradient of proportion of agriculture and landscape diversity. The order of the landscapes presented here in reading sequence corresponds to the geographical order from the northwest to the southeast.

**B - Network sampling protocol**

At each sampling unit, a regular hexagon with 25 m sides was delimited. The center of the hexagon and the six vertices were marked with 1.7 m polyethylene pipes (Figure S2 A). Stripes of yellow/white zebra-striped tape were used to improve the visibility of the pipes from a distance. Beginning from the center, the collectors walked from one pipe to another, linking the center to one vertex of the hexagon, then from this vertex to another, then to the center again, closing an isosceles triangle. This procedure was repeated with the next and succeeding triangles during the 10 hours sampling period, between 7:30 and 17:30 hours, completing as many laps around the hexagon as possible (Figure S2 B to G). During this walk, each of the two collectors followed opposite directions and made short stops (<10 minutes) for observation along the trajectory when flowering plants were sighted; all insects seen visiting flowers inside the hexagon were collected (6) with entomological nets and sacrificed with ethyl acetate for posterior identification in laboratory. Use of ethyl acetate is important to promote the extension of proboscis necessary for the identification of Hymenoptera Aculeata. The sampling was restricted to the range of the entomological nets, which were composed of a cable 1.2 m, a ring of 0.4 m diameter and a conical bag of 1 m deep. At the end of the day, samples of the visited plants were collected and made into herbarium specimens. The insects were deposited at the Museu de Zoologia da Universidade Federal da Bahia (http://www.mzufba.ufba.br/) and the plants samples in the Herbário Alexandre Leal Costa (http://www.alcb.ibio.ufba.br/). Both are located at Universidade Federal da Bahia, Instituto de Biologia, Museu de Zoologia, Campus Universitário, Bairro de Ondina, 40170-115 Salvador, Bahia, Brasil.


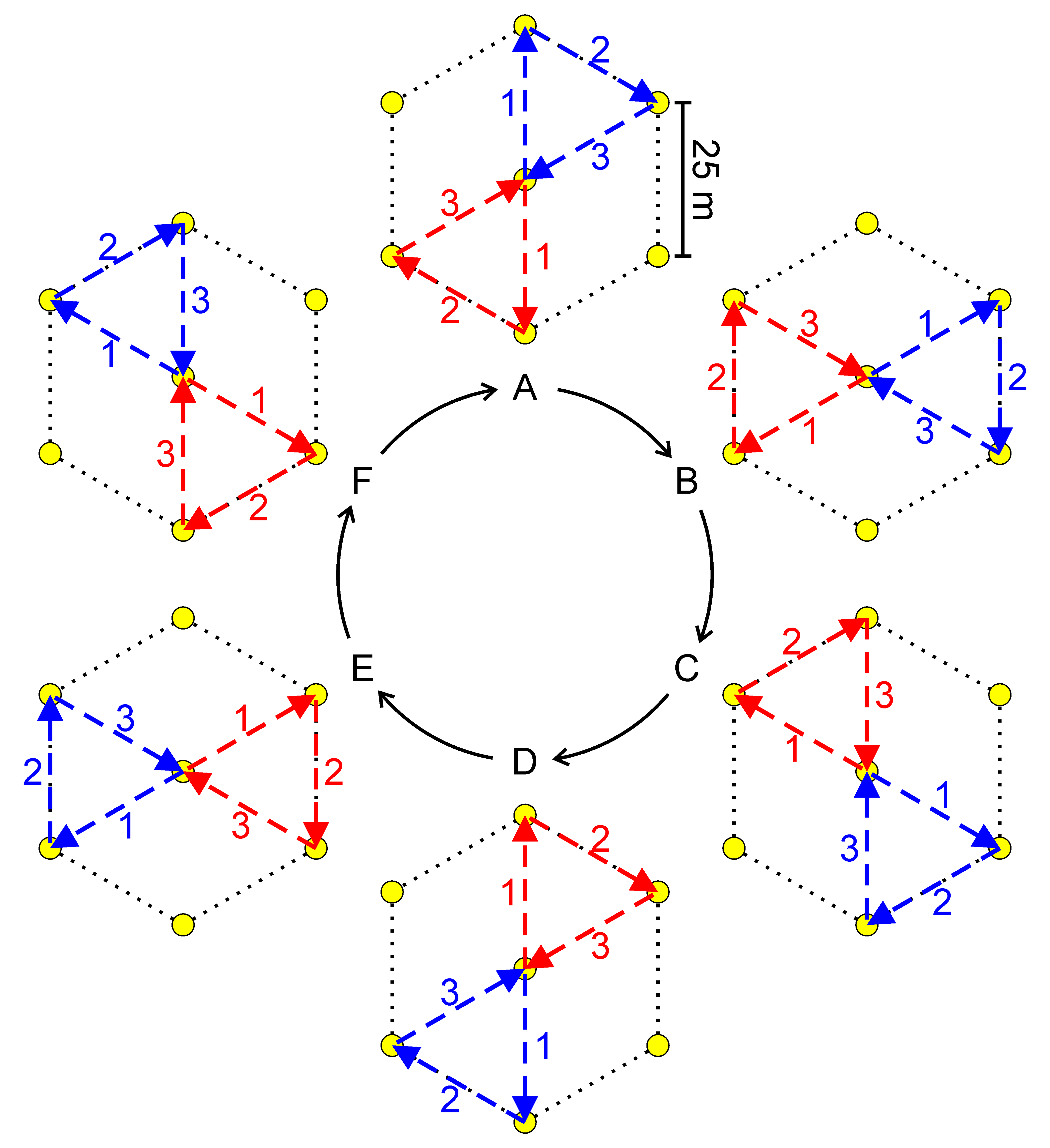


Figure S2 - Scheme of a sampling unit hexagon and movement of the collectors during the sampling; yellow circles represent the flagged polyethylene pipes used for marking the limits of the hexagons; arrows and numbers represent the trajectory and the sequence of motion, respectively, of the two collectors, who are identified by the colors red and blue; A to F – sequence of movements conducted by the collectors during the sampling, covering all sides of the triangles that formed the hexagons.

REFERENCES

1. Hurlbert SH. Pseudoreplication and the Design of Ecological Field Experiments. Ecol Monogr. 1984 Jun;54(2):187.

2. Gathmann A, Tscharntke T. Foraging ranges of solitary bees. J Anim Ecol [Internet]. 2002 Sep;71(5):757–64. Available from: http://doi.wiley.com/10.1046/j.1365-2656.2002.00641.x

3. Steffan-Dewenter I, Münzenberg U, Bürger C, Thies C, Tscharntke T, Hristof CBU, et al. SCALE-DEPENDENT EFFECTS OF LANDSCAPE CONTEXT ON THREE POLLINATOR GUILDS. Ecology. 2002 May;83(5):1421–32.

4. Zurbuchen A, Cheesman S, Klaiber J, Müller A, Hein S, Dorn S. Long foraging distances impose high costs on offspring production in solitary bees. J Anim Ecol. 2010 May;79(3):674–81.

5. Zurbuchen A, Landert L, Klaiber J, Müller A, Hein S, Dorn S. Maximum foraging ranges in solitary bees: only few individuals have the capability to cover long foraging distances. Biol Conserv [Internet]. Elsevier Ltd; 2010 Mar [cited 2014 Oct 1];143(3):669–76. Available from: http://linkinghub.elsevier.com/retrieve/pii/S0006320709005114

6. Sakagami SF, Laroca S, Moure JS. Wild Bee Biocoenotics in São Jose dos Pinhais (PR), South Brazil.：Preliminary Report. 北海道大學理學部紀要 = J Fac Sci HOKKAIDO Univ Ser ⅤⅠ Zool [Internet]. 1967;16(2):253–91. Available from: http://hdl.handle.net/2115/27447
