# Supplementary material for: Spatial Heterogeneity Regulates Plant-Pollinator Networks across Multiple Landscape Scales
Source: PLoS One. 2015 Apr 9;10(4):e0123628. doi: 10.1371/journal.pone.0123628 (PMC4391788; doi:10.1371/journal.pone.0123628)
Supplement: S2 Appendix — (DOC) [file pone.0123628.s002.doc]

**Landscape heterogeneity moderates plant-pollinator networks: a multiple scale approach**

Eduardo Freitas Moreira^1^*, Danilo Boscolo^2^, Blandina Felipe Viana^1^

1 Zoology Department, Federal University of Bahia, UFBA, Salvador, Bahia, Brazil

2 Faculty of Philosophy, Sciences and Literature of Ribeirão Preto, University of São Paulo, Ribeirão Preto, FFCLRP-USP São Paulo, Brazil

* eduardofreitasmoreira@gmail.com

**S2 Appendix– Details of the comparison between the complete plant-pollinator networks and after the exclusion of *Apis mellifera* Linnaeus (1758)**

The model selection procedures showed qualitative differences of plant-pollinator networks if compared before and after the exclusion of the exotic invasive species *A. mellifera*. The null model was among the set of equally plausible models (AICc<2) for nestedness and network interaction strength asymmetry of the complete networks. However, after the exclusion of *A. mellifera*, the null model was never among the most plausible ones (Table A; see also supporting information, Tables S1 to S6). In addition, the ratio between the weight of evidence of the best model and the weight of evidence of the null model increased drastically for all network metrics after the exclusion of *A. mellifera*, indicating that the results became more reliable without this exotic invasive species. These differences in the results of model selection confirmed the assumption that the exotic species *A. mellifera* may mask some responses of native species, especially regarding weighted metrics. In addition, the relationships found in this study were stronger when the exotic species *A. mellifera* was removed from the networks, suggesting that native species are more sensitive to the effects of landscape change (1,2). This finding becomes even more important if we consider that no single-species management policy is able to replace the service provided by the diverse community of native floral visitors observed in the region (3).

**Table A. Summary of model selection for each dependent variable, showing the models with ΔAICc <2 and the subsequent model.**

| **Network metric** | **Hyp.** | **Model** | **∆AICc** | **AICcWi** | **Wi/Wk** |
| --- | --- | --- | --- | --- | --- |
| **Number of interactions complete** | Hyp. 4 | ***y = β_0_ + β_1_ LV + β_2_ PLD*** | 0 | 0.26 | 35.3 |
|  | Hyp. 2 | ***y = β_0_ + β_1_ PLD*** | 1.4 | 0.13 | 17.6 |
|  | Hyp. 4 | ***y = β_0_ + β_1_ LV + β_2_ PLD + β_3_ BLC*** | 2.9 | 0.06 | 8.3 |
| **Number of interactions without *A. mellifera*** | Hyp. 4 | ***y = β_0_ + β_1_ LV + β_2_ PLD*** | 0 | 0.23 | 75.2 |
|  | Hyp. 4 | ***y = β_0_ + β_1_ PLD + β_2_ BLC*** | 0.9 | 0.15 | 47.7 |
|  | Hyp. 2 | ***y = β_0_ + β_1_ PLD*** | 1.2 | 0.13 | 41.1 |
|  | Hyp. 4 | ***y = β_0_ + β_1_ LV + β_2_ PLD + β_3_ BLC*** | 1.7 | 0.1 | 31.4 |
|  | Hyp. 4 | ***y = β_0_ + β_1_ LV + β_2_ PLD + β_3_ BLD*** | 2 | 0.08 | 27.2 |
| **Nestedness complete** | Hyp. 4 | ***y = β_0_ + β_1_ PLD + β_2_ BLD*** | 0 | 0.12 | 1.7 |
|  | Hyp. 3 | ***y = β_0_ + β_1_ BPA + β_2_ BLD*** | 0.3 | 0.11 | 1.5 |
|  | Hyp. 3 | ***y = β_0_ + β_1_ BLD*** | 0.7 | 0.09 | 1.2 |
|  | Hyp. 5 | ***y = β_0_*** | 1.1 | 0.07 | 1 |
|  | Hyp. 3 | ***y = β_0_ + β_1_ BLC + β_2_ BLD*** | 1.1 | 0.07 | - |
|  | Hyp. 3 | ***y = β_0_ + β_1_ BPA + β_2_ BLC + β_3_ BLD*** | 1.7 | 0.05 | - |
|  | Hyp. 2 | ***y = β_0_ + β_1_ PLD*** | 2.6 | 0.03 | - |
| **Nestedness without *A. mellifera*** | Hyp. 4 | ***y = β_0_ + β_1_ PLC + β_2_ BLD*** | 0 | 0.47 | 46.8 |
|  | Hyp. 4 | ***y = β_0_ + β_1_ LV + β_2_ PLC + β_3_ BLD*** | 3 | 0.1 | 10.2 |
| **Network strength asymmetry complete** | Hyp. 2 | ***y = β_0_ + β_1_ PLC*** | 0 | 0.14 | 1.9 |
|  | Hyp. 5 | ***y = β_0_*** | 1.3 | 0.07 | 1 |
|  | Hyp. 4 | ***y = β_0_ + β_1_ LV + β_2_ PLC*** | 1.8 | 0.06 | 0.8 |
|  | Hyp. 3 | ***y = β_0_ + β_1_ BPA*** | 2 | 0.05 | 0.7 |
| **Network strength asymmetry without *A. mellifera*** | Hyp. 4 | ***y = β_0_ + β_1_ PLD + β_2_ BLD*** | 0 | 0.35 | 22.7 |
|  | Hyp. 3 | ***y = β_0_ + β_1_ BLD*** | 2 | 0.13 | 8.3 |

ΔAICc - differences in AICc relative to the lowest value of AICc of all models; AICcW_i_ - Akaike weight of model i; W_i_ / W_k_ - ratio between the weight of model i and the weight of the null model K; Hyp. 1: local vegetation; Hyp. 2: proximal landscape; Hyp. 3: Broad landscape; Hyp. 4: Combined effect; ***β_0_*** - intercept; ***β_1_***, ***β_2_*** and ***β_3_*** - parameters associated with the respective variables; ***LV*** - local vegetation; ***PPA*** – Proximal landscape proportion of agricultural cover; ***PLC*** - Proximal landscape configuration; ***PLD*** - Proximal landscape diversity; ***BPA*** – Broad landscape proportion of agricultural cover; ***BLC*** - Broad landscape configuration; ***BLD*** - Broad landscape diversity.

REFERENCES

1. Ricketts TH, Regetz J, Steffan-Dewenter I, Cunningham S a, Kremen C, Bogdanski A, et al. Landscape effects on crop pollination services: are there general patterns? Ecol Lett [Internet]. 2008 May [cited 2012 Mar 2];11(5):499–515. Available from: http://www.ncbi.nlm.nih.gov/pubmed/18294214

2. Winfree R, Aguilar R, Vázquez DP, LeBuhn G, Aizen MA. A meta-analysis of bees’ responses to anthropogenic disturbance. Ecology [Internet]. Ecological Society of America; 2009 Jul 24 [cited 2013 Dec 15];90(8):2068–76. Available from: http://dx.doi.org/10.1890/08-1245.1

3. Garibaldi LA, Steffan-Dewenter I, Winfree R, Aizen MA, Bommarco R, Cunningham S a, et al. Wild Pollinators Enhance Fruit Set of Crops Regardless of Honey Bee Abundance. Science (80- ) [Internet]. 2013 Mar 29 [cited 2013 Aug 8];339(6127):1608–11. Available from: http://www.ncbi.nlm.nih.gov/pubmed/23449997
