# Supplementary material for: Spatial Heterogeneity Regulates Plant-Pollinator Networks across Multiple Landscape Scales
Source: PLoS One. 2015 Apr 9;10(4):e0123628. doi: 10.1371/journal.pone.0123628 (PMC4391788; doi:10.1371/journal.pone.0123628)
Supplement: S3 Appendix — (DOC) [file pone.0123628.s003.doc]

**Spatial heterogeneity regulates plant-pollinator networks across multiple landscape scales**

Eduardo Freitas Moreira^1^*, Danilo Boscolo^2^, Blandina Felipe Viana^1^

1 Zoology Department, Federal University of Bahia, UFBA, Salvador, Bahia, Brazil

2 Faculty of Philosophy, Sciences and Literature of Ribeirão Preto, University of São Paulo, Ribeirão Preto, FFCLRP-USP São Paulo, Brazil

* eduardofreitasmoreira@gmail.com

**S3 Appendix - Relationship between the proportion of agriculture and landscape structure**

To better evaluate the effect of human intervention on landscape characteristics, we analyzed the relationship between the proportion of agriculture and landscape heterogeneity. For this, a specific experimental design was used in which 70 points were randomly selected on a square grid that covered the entire studied region, with a minimum distance of 3 km between the points (Figure S3). The indexes of proportion of agriculture, landscape diversity and the area-weighted landscape shape index (landscape configuration) were calculated for each point. These calculations were performed with buffers ranging from 250 to 12,000 m. The relationships between the above-cited factors were evaluated using second-order polynomial regression models. The relationships were evaluated only for the variables included in the selected models during the model selection procedure (see Table 2 in the main text). The selection of sampling units and the calculation of the landscape indexes were performed using the module Patch Analyst © Queens Press, Ontario Ministry of Natural Resources, 2012 in ArcGIS 9.3 ESRI® 2008. The regression analysis were performed in R version 2.15.0. The results of the tested relationships are synthesized in Figure S4.
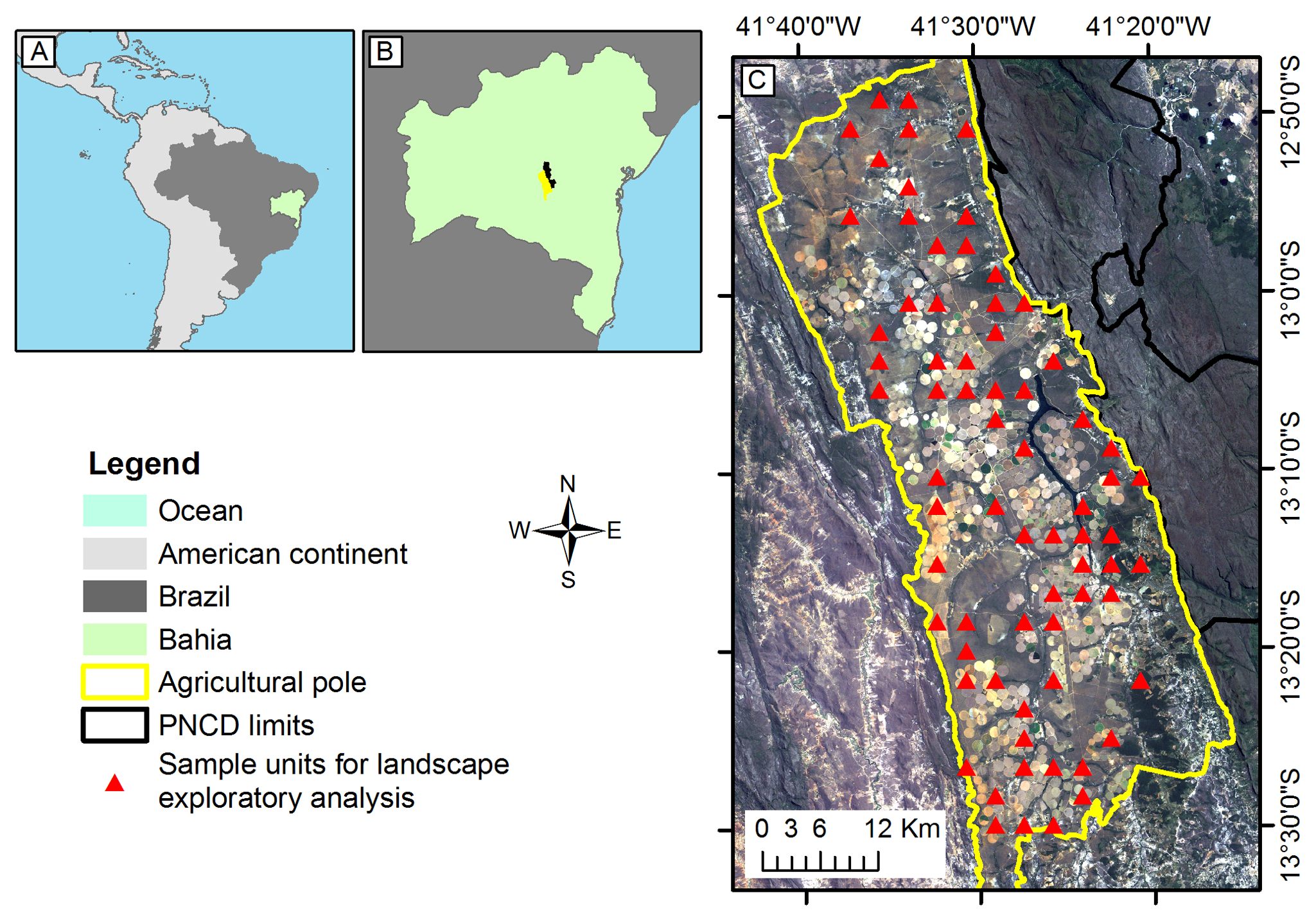
Figure S3 – Distribution of sampling units in the studied region; A - highlighted in green, dark gray and light gray, the state of Bahia, Brazil and South America, respectively; B – At the center of the state of Bahia (green), the studied region, with the agricultural lands in yellow and the Chapada Diamantina National Park in black (Parque Nacional da Chapada Diamantina in Portuguese); C – the 70 sampling units (red triangles) randomly distributed within the boundary of the agricultural pole (yellow); in black, the boundary of Chapada Diamantina National Park.


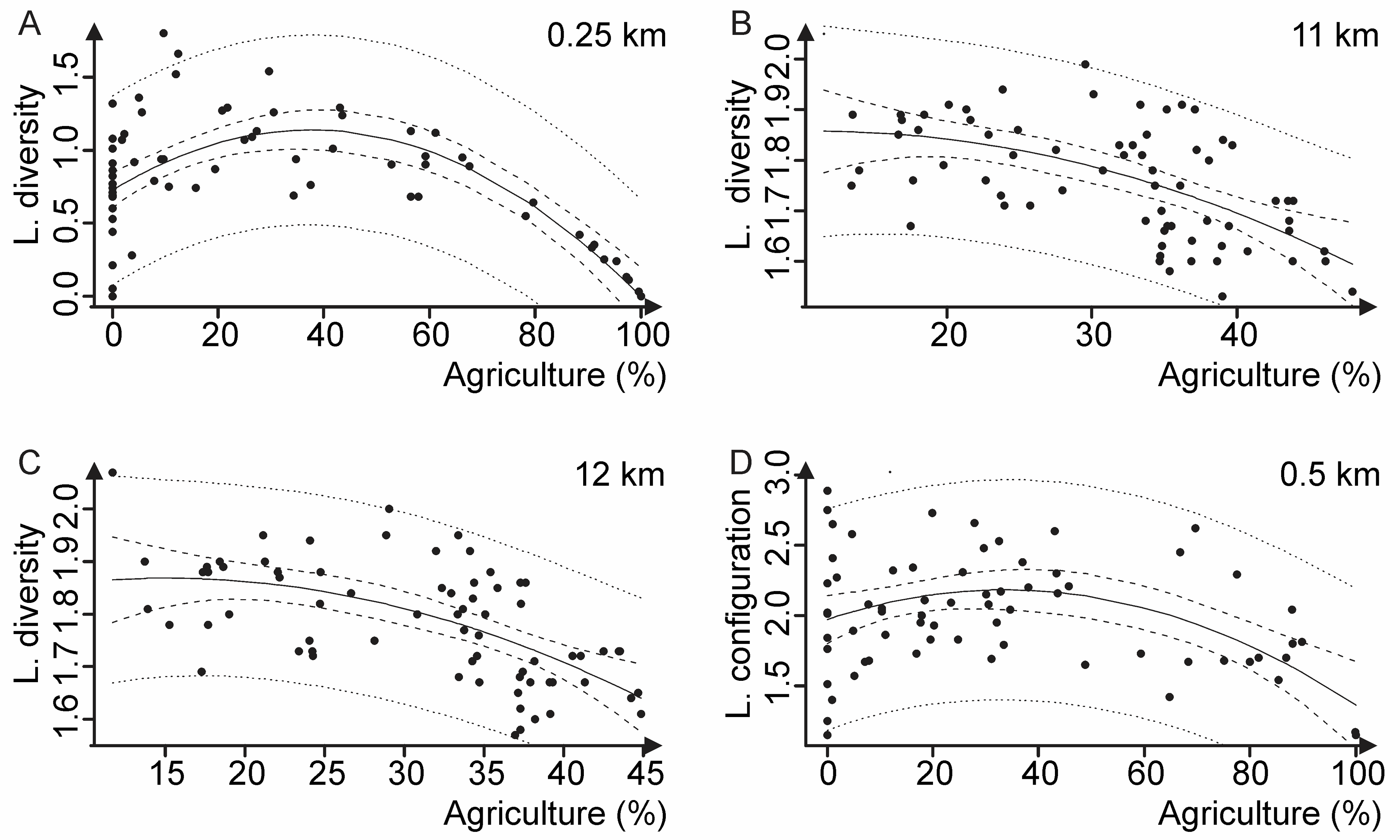


Figure S4 – A – relationship between proportion of agriculture (x axis) and landscape diversity (y axis) at 250 m (df = 67, R2 = 0.52, P = <0.0001); B – relationship between proportion of agriculture (x axis) and landscape diversity (y axis) at 11,000 m (df = 67, R2 = 0.35, P = <0.0001); C – relationship between proportion of agriculture (x axis) and landscape diversity (y axis), at 12,000 m (df = 67, R2 = 0.36, P = <0.0001); D – relationship between proportion of agriculture (x axis) and landscape configuration (y axis) at 500 m (df = 67, R2 = 0.22, P = 0.0002); at the upper right corner of each graph is the measurement scale of the landscape metrics; dots are the sampling units; lines represent the predicted values; dashed lines are the 95% confidence intervals; dotted lines are the 95% prediction intervals.
