# Supplementary material for: Spatial Heterogeneity Regulates Plant-Pollinator Networks across Multiple Landscape Scales
Source: PLoS One. 2015 Apr 9;10(4):e0123628. doi: 10.1371/journal.pone.0123628 (PMC4391788; doi:10.1371/journal.pone.0123628)
Supplement: S2 Table — (DOC) [file pone.0123628.s006.doc]

**Spatial heterogeneity regulates plant-pollinator networks across multiple landscape scales**

Eduardo Freitas Moreira^1^*, Danilo Boscolo^2^, Blandina Felipe Viana^1^

1 Zoology Department, Federal University of Bahia, UFBA, Salvador, Bahia, Brazil

2 Faculty of Philosophy, Sciences and Literature of Ribeirão Preto, University of São Paulo, Ribeirão Preto, FFCLRP-USP São Paulo, Brazil

* eduardofreitasmoreira@gmail.com

**S2 Table:** Model selection ranking for number of interactions with the complete networks.

| **Order** | **Model group** | **Model** | **AICc** | **AICc∆i** | **AICcWi** | **W1/Wi** |
| --- | --- | --- | --- | --- | --- | --- |
| 1 | G4 | y = β0 + β1 LV + β2 PLD | 222.4 | 0 | 0.258 | 1 |
| 2 | G2 | y = β0 + β1 PLD | 223.8 | 1.4 | 0.129 | 2 |
| 3 | G4 | y = β0 + β1 LV + β2 PLD + β3 BLC | 225.3 | 2.9 | 0.06 | 4.3 |
| 4 | G4 | y = β0 + β1 PLD + β2 BLC | 225.4 | 3 | 0.058 | 4.5 |
| 5 | G4 | y = β0 + β1 LV + β2 PLD + β3 BPA | 225.4 | 3 | 0.057 | 4.5 |
| 6 | G4 | y = β0 + β1 LV + β2 PLD + β3 BLD | 225.4 | 3 | 0.057 | 4.6 |
| 7 | G2 | y = β0 + β1 PLC + β2 PLD | 225.6 | 3.2 | 0.052 | 5 |
| 8 | G4 | y = β0 + β1 PLD + β2 BLD | 226.4 | 4 | 0.036 | 7.3 |
| 9 | G2 | y = β0 + β1 PLC | 226.4 | 4 | 0.034 | 7.5 |
| 10 | G4 | y = β0 + β1 LV + β2 PLC | 226.5 | 4.1 | 0.034 | 7.7 |
| 11 | G4 | y = β0 + β1 PLD + β2 BPA | 226.5 | 4.1 | 0.033 | 7.8 |
| 12 | G2 | y = β0 + β1 PPA + β2 PLD | 226.5 | 4.1 | 0.032 | 8 |
| 13 | G4 | y = β0 + β1 PLC + β2 BLC | 227.6 | 5.2 | 0.019 | 13.8 |
| 14 | G4 | y = β0 + β1 LV + β2 PLC + β3 BLD | 228.2 | 5.8 | 0.014 | 18.2 |
| 15 | G4 | y = β0 + β1 PLC + β2 BPA | 228.4 | 6 | 0.013 | 20.3 |
| 16 | G2 | y = β0 + β1 PPA + β2 PLC + β3 PLD | 228.6 | 6.2 | 0.011 | 22.7 |
| 17 | G4 | y = β0 + β1 LV + β2 PLC + β3 BPA | 228.7 | 6.3 | 0.011 | 22.9 |
| 18 | G4 | y = β0 + β1 PLC + β2 BLD | 228.8 | 6.4 | 0.01 | 24.7 |
| 19 | G4 | y = β0 + β1 LV + β2 PLC + β3 BLC | 229.1 | 6.7 | 0.009 | 27.9 |
| 20 | G2 | y = β0 + β1 PPA + β2 PLC | 229.1 | 6.7 | 0.009 | 28.5 |
| 21 | G1 | y = β0 + β1 LV | 229.4 | 7 | 0.008 | 33.1 |
| 22 | Null model | y = β0 | 229.5 | 7.1 | 0.007 | 35.3 |
| 23 | G4 | y = β0 + β1 LV + β2 BPA | 230 | 7.6 | 0.006 | 44.5 |
| 24 | G3 | y = β0 + β1 BPA | 230 | 7.6 | 0.006 | 44.6 |
| 25 | G4 | y = β0 + β1 LV + β2 BLD | 230.7 | 8.3 | 0.004 | 63.6 |
| 26 | G3 | y = β0 + β1 BPA + β2 BLC | 230.9 | 8.5 | 0.004 | 68.8 |
| 27 | G4 | y = β0 + β1 LV + β2 PPA | 230.9 | 8.5 | 0.004 | 69.4 |
| 28 | G2 | y = β0 + β1 PPA | 231 | 8.6 | 0.004 | 71.9 |
| 29 | G3 | y = β0 + β1 BLC | 231.4 | 9 | 0.003 | 90.6 |
| 30 | G4 | y = β0 + β1 LV + β2 PPA + β3 BLD | 231.5 | 9.1 | 0.003 | 96.3 |
| 31 | G3 | y = β0 + β1 BLD | 231.6 | 9.2 | 0.003 | 98.5 |
| 32 | G4 | y = β0 + β1 PPA + β2 BPA | 231.8 | 9.4 | 0.002 | 110.8 |
| 33 | G4 | y = β0 + β1 LV + β2 PPA + β3 BPA | 231.9 | 9.5 | 0.002 | 116.3 |
| 34 | G4 | y = β0 + β1 LV + β2 BLC | 232.1 | 9.7 | 0.002 | 129.7 |
| 35 | G3 | y = β0 + β1 BPA + β2 BLD | 232.8 | 10.4 | 0.001 | 178 |
| 36 | G4 | y = β0 + β1 PPA + β2 BLD | 232.8 | 10.4 | 0.001 | 181.8 |
| 37 | G4 | y = β0 + β1 PPA + β2 BLC | 233.1 | 10.7 | 0.001 | 208.2 |
| 38 | G3 | y = β0 + β1 BLC + β2 BLD | 233.2 | 10.8 | 0.001 | 216.9 |
| 39 | G3 | y = β0 + β1 BPA + β2 BLC + β3 BLD | 233.8 | 11.4 | <0.001 | >217 |
| 40 | G4 | y = β0 + β1 LV + β2 PPA + β3 BLC | 233.9 | 11.5 | <0.001 | >217 |

AICcΔ - differences in AICc relative to the lowest value of AICc of all models; AICcW_i_ - Akaike weight of model i; W_1_ / W_i_ - ratio between the weight of model 1 and the weight of the respective model; G1 - Local vegetation; G2 - Proximal landscape structure; G3 - Broad landscape structure; G4 Multi-level combined effect; Null model – no effect; *β_0_* - intercept; *β_1_*, *β_2_* and *β_3_* - parameters associated with the respective variables; *LV* - local vegetation; *PPA* – Proximal landscape proportion of agricultural cover; *PLC* - Proximal landscape configuration; *PLD* - Proximal landscape diversity; *BPA* – Broad landscape proportion of agricultural cover; *BLC* - Broad landscape configuration; *BLD* - Broad landscape diversity.
