# Supplementary material for: Spatial Heterogeneity Regulates Plant-Pollinator Networks across Multiple Landscape Scales
Source: PLoS One. 2015 Apr 9;10(4):e0123628. doi: 10.1371/journal.pone.0123628 (PMC4391788; doi:10.1371/journal.pone.0123628)
Supplement: S3 Table — (DOC) [file pone.0123628.s007.doc]

**Spatial heterogeneity regulates plant-pollinator networks across multiple landscape scales**

Eduardo Freitas Moreira^1^*, Danilo Boscolo^2^, Blandina Felipe Viana^1^

1 Zoology Department, Federal University of Bahia, UFBA, Salvador, Bahia, Brazil

2 Faculty of Philosophy, Sciences and Literature of Ribeirão Preto, University of São Paulo, Ribeirão Preto, FFCLRP-USP São Paulo, Brazil

* eduardofreitasmoreira@gmail.com

**S3 Table:** Model selection ranking for number of interactions without the species *Apis mellifera* Linnaeus (1758).

| **Order** | **Model group** | **Model** | **AICc** | **AICc∆i** | **AICcWi** | **W1/Wi** |
| --- | --- | --- | --- | --- | --- | --- |
| 1 | G4 | *y = β_0_ + β_1_ LV + β_2_ PLD* | 212.3 | 0 | 0.232 | 1 |
| 2 | G4 | *y = β_0_ + β_1_ PLD + β_2_ BLC* | 213.2 | 0.9 | 0.147 | 1.6 |
| 3 | G2 | *y = β_0_ + β_1_ PLD* | 213.5 | 1.2 | 0.127 | 1.8 |
| 4 | G4 | *y = β_0_ + β_1_ LV + β_2_ PLD + β_3_ BLC* | 214.1 | 1.7 | 0.097 | 2.4 |
| 5 | G4 | *y = β_0_ + β_1_ LV + β_2_ PLD + β_3_ BLD* | 214.3 | 2 | 0.084 | 2.8 |
| 6 | G4 | *y = β_0_ + β_1_ LV + β_2_ PLD + β_3_ BPA* | 215.1 | 2.7 | 0.059 | 4 |
| 7 | G2 | *y = β_0_ + β_1_ PPA + β_2_ PLD* | 215.5 | 3.2 | 0.047 | 4.9 |
| 8 | G4 | *y = β_0_ + β_1_ PLD + β_2_ BPA* | 215.9 | 3.6 | 0.037 | 6.2 |
| 9 | G4 | *y = β_0_ + β_1_ PLD + β_2_ BLD* | 216.1 | 3.8 | 0.035 | 6.7 |
| 10 | G2 | *y = β_0_ + β_1_ PLC + β_2_ PLD* | 216.3 | 4 | 0.032 | 7.3 |
| 11 | G3 | *y = β_0_ + β_1_ BPA + β_2_ BLC* | 218.3 | 6 | 0.012 | 19.7 |
| 12 | G2 | *y = β_0_ + β_1_ PPA + β_2_ PLC + β_3_ PLD* | 218.5 | 6.2 | 0.01 | 22.3 |
| 13 | G3 | *y = β_0_ + β_1_ BPA + β_2_ BLC + β_3_ BLD* | 219.9 | 7.5 | 0.005 | 43.6 |
| 14 | G4 | *y = β_0_ + β_1_ PPA + β_2_ BPA* | 220 | 7.7 | 0.005 | 46.3 |
| 15 | G4 | *y = β_0_ + β_1_ LV + β_2_ PPA + β_3_ BPA* | 220.1 | 7.8 | 0.005 | 48.5 |
| 16 | G3 | *y = β_0_ + β_1_ BPA* | 220.1 | 7.8 | 0.005 | 49.8 |
| 17 | G4 | *y = β_0_ + β_1_ PLC + β_2_ BLC* | 220.2 | 7.8 | 0.005 | 50.6 |
| 18 | G2 | *y = β_0_ + β_1_ PLC* | 220.2 | 7.9 | 0.004 | 51.6 |
| 19 | G4 | *y = β_0_ + β_1_ LV + β_2_ BPA* | 220.4 | 8.1 | 0.004 | 56.2 |
| 20 | G2 | *y = β_0_ + β_1_ PPA* | 220.4 | 8.1 | 0.004 | 57.2 |
| 21 | G4 | *y = β_0_ + β_1_ LV + β_2_ PPA* | 220.4 | 8.1 | 0.004 | 58.1 |
| 22 | G4 | *y = β_0_ + β_1_ LV + β_2_ PLC* | 220.7 | 8.4 | 0.004 | 66.2 |
| 23 | G4 | *y = β_0_ + β_1_ PLC + β_2_ BPA* | 220.9 | 8.6 | 0.003 | 74.3 |
| 24 | Null model | *y = β_0_* | 220.9 | 8.6 | 0.003 | 75.2 |
| 25 | G1 | *y = β_0_ + β_1_ LV* | 221.1 | 8.8 | 0.003 | 82.8 |
| 26 | G3 | *y = β_0_ + β_1_ BLC + β_2_ BLD* | 221.2 | 8.9 | 0.003 | 84.6 |
| 27 | G4 | *y = β_0_ + β_1_ PPA + β_2_ BLC* | 221.3 | 9 | 0.003 | 90.9 |
| 28 | G2 | *y = β_0_ + β_1_ PPA + β_2_ PLC* | 221.4 | 9.1 | 0.003 | 92.7 |
| 29 | G4 | *y = β_0_ + β_1_ LV + β_2_ PPA + β_3_ BLD* | 221.4 | 9.1 | 0.002 | 93.8 |
| 30 | G4 | *y = β_0_ + β_1_ LV + β_2_ PLC + β_3_ BLD* | 221.5 | 9.2 | 0.002 | 97.8 |
| 31 | G4 | *y = β_0_ + β_1_ LV + β_2_ PLC + β_3_ BPA* | 221.5 | 9.2 | 0.002 | 99.9 |
| 32 | G4 | *y = β_0_ + β_1_ LV + β_2_ BLD* | 221.7 | 9.4 | 0.002 | 108.3 |
| 33 | G3 | *y = β_0_ + β_1_ BLC* | 221.8 | 9.5 | 0.002 | 113 |
| 34 | G4 | *y = β_0_ + β_1_ PLC + β_2_ BLD* | 221.9 | 9.6 | 0.002 | 122.6 |
| 35 | G4 | *y = β_0_ + β_1_ LV + β_2_ PLC + β_3_ BLC* | 222.3 | 10 | 0.002 | 146.7 |
| 36 | G4 | *y = β_0_ + β_1_ PPA + β_2_ BLD* | 222.3 | 10 | 0.002 | 149.5 |
| 37 | G3 | *y = β_0_ + β_1_ BLD* | 222.5 | 10.2 | 0.001 | 162 |
| 38 | G4 | *y = β_0_ + β_1_ LV + β_2_ PPA + β_3_ BLC* | 222.8 | 10.5 | 0.001 | 189.9 |
| 39 | G3 | *y = β_0_ + β_1_ BPA + β_2_ BLD* | 222.8 | 10.5 | 0.001 | 189.9 |
| 40 | G4 | *y = β_0_ + β_1_ LV + β_2_ BLC* | 223.2 | 10.9 | 0.001 | 257.5 |

AICcΔ - differences in AICc relative to the lowest value of AICc of all models; AICcW_i_ - Akaike weight of model i; W_1_ / W_i_ - ratio between the weight of model 1 and the weight of the respective model; G1 - Local vegetation; G2 - Proximal landscape structure; G3 - Broad landscape structure; G4 Multi-level combined effect; Null model – no effect; *β_0_* - intercept; *β_1_*, *β_2_* and *β_3_* - parameters associated with the respective variables; *LV* - local vegetation; *PPA* – Proximal landscape proportion of agricultural cover; *PLC* - Proximal landscape configuration; *PLD* - Proximal landscape diversity; *BPA* – Broad landscape proportion of agricultural cover; *BLC* - Broad landscape configuration; *BLD* - Broad landscape diversity.
