# Supplementary material for: Spatial Heterogeneity Regulates Plant-Pollinator Networks across Multiple Landscape Scales
Source: PLoS One. 2015 Apr 9;10(4):e0123628. doi: 10.1371/journal.pone.0123628 (PMC4391788; doi:10.1371/journal.pone.0123628)
Supplement: S4 Table — (DOC) [file pone.0123628.s008.doc]

**Spatial heterogeneity regulates plant-pollinator networks across multiple landscape scales**

Eduardo Freitas Moreira^1^*, Danilo Boscolo^2^, Blandina Felipe Viana^1^

1 Zoology Department, Federal University of Bahia, UFBA, Salvador, Bahia, Brazil

2 Faculty of Philosophy, Sciences and Literature of Ribeirão Preto, University of São Paulo, Ribeirão Preto, FFCLRP-USP São Paulo, Brazil

* eduardofreitasmoreira@gmail.com

**S4 Table:** Model selection ranking for network nestedness with the complete networks.

| **Order** | **Model group** | **Model** | **AICc** | **AICc∆i** | **AICcWi** | **W1/Wi** |
| --- | --- | --- | --- | --- | --- | --- |
| 1 | G4 | *y = β_0_ + β_1_ PLD + β_2_ BLD* | 165.8 | 0 | 0.124 | 1 |
| 2 | G3 | *y = β_0_ + β_1_ BPA + β_2_ BLD* | 166.1 | 0.3 | 0.108 | 1.1 |
| 3 | G3 | *y = β_0_ + β_1_ BLD* | 166.5 | 0.7 | 0.086 | 1.4 |
| 4 | Null model | *y = β_0_* | 166.9 | 1.1 | 0.072 | 1.7 |
| 5 | G3 | *y = β_0_ + β_1_ BLC + β_2_ BLD* | 166.9 | 1.1 | 0.072 | 1.7 |
| 6 | G3 | *y = β_0_ + β_1_ BPA + β_2_ BLC + β_3_ BLD* | 167.5 | 1.7 | 0.053 | 2.3 |
| 7 | G2 | *y = β_0_ + β_1_ PLD* | 168.4 | 2.6 | 0.034 | 3.7 |
| 8 | G1 | *y = β_0_ + β_1_ LV* | 168.5 | 2.7 | 0.033 | 3.8 |
| 9 | G4 | *y = β_0_ + β_1_ PLC + β_2_ BLD* | 168.5 | 2.7 | 0.033 | 3.8 |
| 10 | G4 | *y = β_0_ + β_1_ PPA + β_2_ BLD* | 168.5 | 2.7 | 0.032 | 3.8 |
| 11 | G2 | *y = β_0_ + β_1_ PPA* | 168.6 | 2.7 | 0.031 | 3.9 |
| 12 | G2 | *y = β_0_ + β_1_ PLC* | 168.6 | 2.8 | 0.03 | 4.1 |
| 13 | G4 | *y = β_0_ + β_1_ LV + β_2_ PLD + β_3_ BLD* | 168.8 | 3 | 0.027 | 4.5 |
| 14 | G3 | *y = β_0_ + β_1_ BPA* | 168.9 | 3.1 | 0.027 | 4.6 |
| 15 | G4 | *y = β_0_ + β_1_ LV + β_2_ BLD* | 169.3 | 3.4 | 0.022 | 5.6 |
| 16 | G3 | *y = β_0_ + β_1_ BLC* | 169.3 | 3.5 | 0.021 | 5.8 |
| 17 | G4 | *y = β_0_ + β_1_ LV + β_2_ PLC* | 170.1 | 4.3 | 0.015 | 8.5 |
| 18 | G4 | *y = β_0_ + β_1_ LV + β_2_ PLD* | 170.3 | 4.4 | 0.013 | 9.2 |
| 19 | G4 | *y = β_0_ + β_1_ LV + β_2_ PPA* | 170.4 | 4.6 | 0.012 | 10.1 |
| 20 | G4 | *y = β_0_ + β_1_ LV + β_2_ BPA* | 170.5 | 4.7 | 0.012 | 10.3 |
| 21 | G4 | *y = β_0_ + β_1_ PLC + β_2_ BPA* | 170.5 | 4.7 | 0.012 | 10.6 |
| 22 | G2 | *y = β_0_ + β_1_ PLC + β_2_ PLD* | 170.9 | 5 | 0.01 | 12.4 |
| 23 | G4 | *y = β_0_ + β_1_ LV + β_2_ BLC* | 170.9 | 5 | 0.01 | 12.5 |
| 24 | G2 | *y = β_0_ + β_1_ PPA + β_2_ PLC* | 171 | 5.1 | 0.009 | 13 |
| 25 | G2 | *y = β_0_ + β_1_ PPA + β_2_ PLD* | 171 | 5.1 | 0.009 | 13.1 |
| 26 | G4 | *y = β_0_ + β_1_ PLD + β_2_ BPA* | 171 | 5.2 | 0.009 | 13.5 |
| 27 | G4 | *y = β_0_ + β_1_ PPA + β_2_ BPA* | 171.1 | 5.3 | 0.009 | 14 |
| 28 | G4 | *y = β_0_ + β_1_ PLD + β_2_ BLC* | 171.2 | 5.3 | 0.009 | 14.5 |
| 29 | G4 | *y = β_0_ + β_1_ PPA + β_2_ BLC* | 171.2 | 5.4 | 0.008 | 14.9 |
| 30 | G4 | *y = β_0_ + β_1_ PLC + β_2_ BLC* | 171.2 | 5.4 | 0.008 | 15 |
| 31 | G4 | *y = β_0_ + β_1_ LV + β_2_ PLC + β_3_ BLD* | 171.3 | 5.5 | 0.008 | 15.8 |
| 32 | G4 | *y = β_0_ + β_1_ LV + β_2_ PPA + β_3_ BLD* | 171.5 | 5.7 | 0.007 | 17 |
| 33 | G3 | *y = β_0_ + β_1_ BPA + β_2_ BLC* | 171.7 | 5.8 | 0.007 | 18.5 |
| 34 | G4 | *y = β_0_ + β_1_ LV + β_2_ PLC + β_3_ BPA* | 171.9 | 6 | 0.006 | 20.6 |
| 35 | G4 | *y = β_0_ + β_1_ LV + β_2_ PLC + β_3_ BLC* | 172.5 | 6.7 | 0.004 | 28.7 |
| 36 | G4 | *y = β_0_ + β_1_ LV + β_2_ PLD + β_3_ BPA* | 173 | 7.2 | 0.003 | 35.8 |
| 37 | G4 | *y = β_0_ + β_1_ LV + β_2_ PLD + β_3_ BLC* | 173.1 | 7.3 | 0.003 | 37.5 |
| 38 | G4 | *y = β_0_ + β_1_ LV + β_2_ PPA + β_3_ BPA* | 173.1 | 7.3 | 0.003 | 37.7 |
| 39 | G4 | *y = β_0_ + β_1_ LV + β_2_ PPA + β_3_ BLC* | 173.1 | 7.3 | 0.003 | 38 |
| 40 | G2 | *y = β_0_ + β_1_ PPA + β_2_ PLC + β_3_ PLD* | 173.8 | 7.9 | 0.002 | 52.9 |

AICcΔ - differences in AICc relative to the lowest value of AICc of all models; AICcW_i_ - Akaike weight of model i; W_1_ / W_i_ - ratio between the weight of model 1 and the weight of the respective model; G1 - Local vegetation; G2 - Proximal landscape structure; G3 - Broad landscape structure; G4 Multi-level combined effect; Null model – no effect; *β_0_* - intercept; *β_1_*, *β_2_* and *β_3_* - parameters associated with the respective variables; *LV* - local vegetation; *PPA* – Proximal landscape proportion of agricultural cover; *PLC* - Proximal landscape configuration; *PLD* - Proximal landscape diversity; *BPA* – Broad landscape proportion of agricultural cover; *BLC* - Broad landscape configuration; *BLD* - Broad landscape diversity.
