# Supplementary material for: Spatial Heterogeneity Regulates Plant-Pollinator Networks across Multiple Landscape Scales
Source: PLoS One. 2015 Apr 9;10(4):e0123628. doi: 10.1371/journal.pone.0123628 (PMC4391788; doi:10.1371/journal.pone.0123628)
Supplement: S5 Table — (DOC) [file pone.0123628.s009.doc]

**Spatial heterogeneity regulates plant-pollinator networks across multiple landscape scales**

Eduardo Freitas Moreira^1^*, Danilo Boscolo^2^, Blandina Felipe Viana^1^

1 Zoology Department, Federal University of Bahia, UFBA, Salvador, Bahia, Brazil

2 Faculty of Philosophy, Sciences and Literature of Ribeirão Preto, University of São Paulo, Ribeirão Preto, FFCLRP-USP São Paulo, Brazil

* eduardofreitasmoreira@gmail.com

**S5 Table:** Model selection ranking for network nestedness without the species *Apis mellifera* Linnaeus (1758).

| **Order** | **Model group** | **Model** | **AICc** | **AICc∆i** | **AICcWi** | **W1/Wi** |
| --- | --- | --- | --- | --- | --- | --- |
| 1 | G4 | *y = β_0_ + β_1_ PLC + β_2_ BLD* | 172 | 0 | 0.467 | 1 |
| 2 | G4 | *y = β_0_ + β_1_ LV + β_2_ PLC + β_3_ BLD* | 175.1 | 3 | 0.102 | 4.6 |
| 3 | G2 | *y = β_0_ + β_1_ PLC* | 176.1 | 4 | 0.063 | 7.4 |
| 4 | G4 | *y = β_0_ + β_1_ PPA + β_2_ BLD* | 176.4 | 4.4 | 0.052 | 9 |
| 5 | G3 | *y = β_0_ + β_1_ BPA + β_2_ BLD* | 176.6 | 4.6 | 0.047 | 10 |
| 6 | G4 | *y = β_0_ + β_1_ LV + β_2_ PLC* | 177.5 | 5.4 | 0.031 | 15.2 |
| 7 | G4 | *y = β_0_ + β_1_ PLC + β_2_ BLC* | 177.8 | 5.8 | 0.026 | 17.9 |
| 8 | G3 | *y = β_0_ + β_1_ BLD* | 178 | 6 | 0.024 | 19.7 |
| 9 | G2 | *y = β_0_ + β_1_ PPA + β_2_ PLC* | 178.3 | 6.2 | 0.021 | 22.5 |
| 10 | G2 | *y = β_0_ + β_1_ PLC + β_2_ PLD* | 178.5 | 6.5 | 0.018 | 25.5 |
| 11 | G4 | *y = β_0_ + β_1_ PLC + β_2_ BPA* | 178.8 | 6.8 | 0.016 | 29.6 |
| 12 | G4 | *y = β_0_ + β_1_ LV + β_2_ PPA + β_3_ BLD* | 179.2 | 7.2 | 0.013 | 36.2 |
| 13 | G3 | *y = β_0_ + β_1_ BPA + β_2_ BLC + β_3_ BLD* | 179.6 | 7.5 | 0.011 | 43.2 |
| 14 | Null model | *y = β_0_* | 179.7 | 7.7 | 0.01 | 46.8 |
| 15 | G1 | *y = β_0_ + β_1_ LV* | 180 | 8 | 0.009 | 53.9 |
| 16 | G4 | *y = β_0_ + β_1_ LV + β_2_ PLC + β_3_ BLC* | 180.1 | 8.1 | 0.008 | 57 |
| 17 | G4 | *y = β_0_ + β_1_ LV + β_2_ BLD* | 180.2 | 8.2 | 0.008 | 60.2 |
| 18 | G3 | *y = β_0_ + β_1_ BLC + β_2_ BLD* | 180.4 | 8.3 | 0.007 | 63.8 |
| 19 | G4 | *y = β_0_ + β_1_ LV + β_2_ PLC + β_3_ BPA* | 180.4 | 8.4 | 0.007 | 65.6 |
| 20 | G4 | *y = β_0_ + β_1_ PLD + β_2_ BLD* | 180.7 | 8.7 | 0.006 | 77.8 |
| 21 | G2 | *y = β_0_ + β_1_ PPA + β_2_ PLC + β_3_ PLD* | 180.8 | 8.8 | 0.006 | 80 |
| 22 | G2 | *y = β_0_ + β_1_ PPA* | 180.8 | 8.8 | 0.006 | 81.4 |
| 23 | G4 | *y = β_0_ + β_1_ LV + β_2_ PPA* | 181.2 | 9.1 | 0.005 | 96 |
| 24 | G3 | *y = β_0_ + β_1_ BPA* | 181.4 | 9.4 | 0.004 | 109.7 |
| 25 | G4 | *y = β_0_ + β_1_ LV + β_2_ BPA* | 181.5 | 9.5 | 0.004 | 113.5 |
| 26 | G2 | *y = β_0_ + β_1_ PLD* | 181.7 | 9.7 | 0.004 | 125 |
| 27 | G3 | *y = β_0_ + β_1_ BLC* | 181.9 | 9.9 | 0.003 | 139.5 |
| 28 | G4 | *y = β_0_ + β_1_ LV + β_2_ PLD* | 182.2 | 10.1 | 0.003 | 158.5 |
| 29 | G4 | *y = β_0_ + β_1_ PPA + β_2_ BLC* | 182.6 | 10.5 | 0.002 | 195.6 |
| 30 | G2 | *y = β_0_ + β_1_ PPA + β_2_ PLD* | 182.7 | 10.6 | 0.002 | 204.1 |
| 31 | G4 | *y = β_0_ + β_1_ LV + β_2_ BLC* | 182.8 | 10.7 | 0.002 | 214.4 |
| 32 | G4 | *y = β_0_ + β_1_ PLD + β_2_ BPA* | 183.2 | 11.1 | 0.002 | 261.2 |
| 33 | G4 | *y = β_0_ + β_1_ LV + β_2_ PLD + β_3_ BLD* | 183.2 | 11.2 | 0.002 | 265.6 |
| 34 | G3 | *y = β_0_ + β_1_ BPA + β_2_ BLC* | 183.2 | 11.2 | 0.002 | 268.7 |
| 35 | G4 | *y = β_0_ + β_1_ LV + β_2_ PLD + β_3_ BPA* | 183.3 | 11.3 | 0.002 | 279.9 |
| 36 | G4 | *y = β_0_ + β_1_ PPA + β_2_ BPA* | 183.6 | 11.6 | 0.001 | 324.6 |
| 37 | G4 | *y = β_0_ + β_1_ LV + β_2_ PPA + β_3_ BLC* | 183.9 | 11.9 | 0.001 | 383.2 |
| 38 | G4 | *y = β_0_ + β_1_ LV + β_2_ PPA + β_3_ BPA* | 184.1 | 12.1 | 0.001 | 417.4 |
| 39 | G4 | *y = β_0_ + β_1_ PLD + β_2_ BLC* | 184.3 | 12.2 | 0.001 | 449.5 |
| 40 | G4 | *y = β_0_ + β_1_ LV + β_2_ PLD + β_3_ BLC* | 185.2 | 13.2 | <0.001 | >449.6 |

AICcΔ - differences in AICc relative to the lowest value of AICc of all models; AICcW_i_ - Akaike weight of model i; W_1_ / W_i_ - ratio between the weight of model 1 and the weight of the respective model; G1 - Local vegetation; G2 - Proximal landscape structure; G3 - Broad landscape structure; G4 Multi-level combined effect; Null model – no effect; *β_0_* - intercept; *β_1_*, *β_2_* and *β_3_* - parameters associated with the respective variables; *LV* - local vegetation; *PPA* – Proximal landscape proportion of agricultural cover; *PLC* - Proximal landscape configuration; *PLD* - Proximal landscape diversity; *BPA* – Broad landscape proportion of agricultural cover; *BLC* - Broad landscape configuration; *BLD* - Broad landscape diversity.
