# Supplementary material for: Spatial Heterogeneity Regulates Plant-Pollinator Networks across Multiple Landscape Scales
Source: PLoS One. 2015 Apr 9;10(4):e0123628. doi: 10.1371/journal.pone.0123628 (PMC4391788; doi:10.1371/journal.pone.0123628)
Supplement: S7 Table — (DOC) [file pone.0123628.s011.doc]

**Spatial heterogeneity regulates plant-pollinator networks across multiple landscape scales**

Eduardo Freitas Moreira^1^*, Danilo Boscolo^2^, Blandina Felipe Viana^1^

1 Zoology Department, Federal University of Bahia, UFBA, Salvador, Bahia, Brazil

2 Faculty of Philosophy, Sciences and Literature of Ribeirão Preto, University of São Paulo, Ribeirão Preto, FFCLRP-USP São Paulo, Brazil

* eduardofreitasmoreira@gmail.com

**S8 Table:** Model selection ranking for network interaction strength asymmetry without the species *Apis mellifera* Linnaeus (1758).

| **Order** | **Model group** | **Model** | **AICc** | **AICc∆i** | **AICcWi** | **W1/Wi** |
| --- | --- | --- | --- | --- | --- | --- |
| 1 | G4 | *y = β_0_ + β_1_ PLD + β_2_ BLD* | -19.7 | 0 | 0.352 | 1 |
| 2 | G3 | *y = β_0_ + β_1_ BLD* | -17.7 | 2 | 0.129 | 2.7 |
| 3 | G4 | *y = β_0_ + β_1_ LV + β_2_ PLD + β_3_ BLD* | -16.8 | 2.9 | 0.084 | 4.2 |
| 4 | G4 | *y = β_0_ + β_1_ PLC + β_2_ BLD* | -15.8 | 3.9 | 0.051 | 7 |
| 5 | G4 | *y = β_0_ + β_1_ PPA + β_2_ BLD* | -15.4 | 4.3 | 0.041 | 8.6 |
| 6 | G3 | *y = β_0_ + β_1_ BLC + β_2_ BLD* | -15.2 | 4.5 | 0.038 | 9.3 |
| 7 | G4 | *y = β_0_ + β_1_ LV + β_2_ BLD* | -14.9 | 4.8 | 0.033 | 10.8 |
| 8 | G3 | *y = β_0_ + β_1_ BPA + β_2_ BLD* | -14.9 | 4.8 | 0.032 | 10.9 |
| 9 | G4 | *y = β_0_ + β_1_ PLD + β_2_ BLC* | -14.4 | 5.3 | 0.025 | 14.1 |
| 10 | G3 | *y = β_0_ + β_1_ BLC* | -14 | 5.6 | 0.021 | 16.8 |
| 11 | G4 | *y = β_0_ + β_1_ PLD + β_2_ BPA* | -13.9 | 5.8 | 0.019 | 18.4 |
| 12 | Null model | *y = β_0_* | -13.4 | 6.2 | 0.016 | 22.7 |
| 13 | G4 | *y = β_0_ + β_1_ LV + β_2_ PLC + β_3_ BLD* | -12.8 | 6.9 | 0.011 | 31.2 |
| 14 | G2 | *y = β_0_ + β_1_ PLD* | -12.8 | 6.9 | 0.011 | 31.6 |
| 15 | G4 | *y = β_0_ + β_1_ PLC + β_2_ BLC* | -12.5 | 7.1 | 0.01 | 35.6 |
| 16 | G2 | *y = β_0_ + β_1_ PLC* | -12.5 | 7.2 | 0.01 | 36.9 |
| 17 | G2 | *y = β_0_ + β_1_ PPA* | -12.3 | 7.3 | 0.009 | 39 |
| 18 | G4 | *y = β_0_ + β_1_ LV + β_2_ PPA + β_3_ BLD* | -12.3 | 7.3 | 0.009 | 39.3 |
| 19 | G3 | *y = β_0_ + β_1_ BPA + β_2_ BLC + β_3_ BLD* | -12.2 | 7.5 | 0.008 | 42.2 |
| 20 | G4 | *y = β_0_ + β_1_ LV + β_2_ PLD + β_3_ BPA* | -12.1 | 7.6 | 0.008 | 44.8 |
| 21 | G4 | *y = β_0_ + β_1_ PPA + β_2_ BLC* | -11.9 | 7.8 | 0.007 | 48.9 |
| 22 | G3 | *y = β_0_ + β_1_ BPA* | -11.8 | 7.9 | 0.007 | 51.2 |
| 23 | G1 | *y = β_0_ + β_1_ LV* | -11.7 | 8 | 0.007 | 53.5 |
| 24 | G3 | *y = β_0_ + β_1_ BPA + β_2_ BLC* | -11.5 | 8.2 | 0.006 | 60.5 |
| 25 | G4 | *y = β_0_ + β_1_ LV + β_2_ PLC* | -11.4 | 8.2 | 0.006 | 61.3 |
| 26 | G4 | *y = β_0_ + β_1_ LV + β_2_ PLD + β_3_ BLC* | -11.4 | 8.3 | 0.006 | 62.4 |
| 27 | G4 | *y = β_0_ + β_1_ LV + β_2_ BLC* | -11.4 | 8.3 | 0.006 | 63.1 |
| 28 | G2 | *y = β_0_ + β_1_ PPA + β_2_ PLD* | -11.2 | 8.5 | 0.005 | 70.5 |
| 29 | G2 | *y = β_0_ + β_1_ PLC + β_2_ PLD* | -11.1 | 8.6 | 0.005 | 73.4 |
| 30 | G4 | *y = β_0_ + β_1_ LV + β_2_ PLD* | -10.9 | 8.8 | 0.004 | 82 |
| 31 | G2 | *y = β_0_ + β_1_ PPA + β_2_ PLC* | -10.8 | 8.9 | 0.004 | 86.4 |
| 32 | G4 | *y = β_0_ + β_1_ LV + β_2_ PPA* | -10.5 | 9.1 | 0.004 | 96.8 |
| 33 | G4 | *y = β_0_ + β_1_ PPA + β_2_ BPA* | -10.3 | 9.4 | 0.003 | 108.8 |
| 34 | G4 | *y = β_0_ + β_1_ LV + β_2_ PLC + β_3_ BLC* | -10.1 | 9.6 | 0.003 | 121.1 |
| 35 | G4 | *y = β_0_ + β_1_ PLC + β_2_ BPA* | -10.1 | 9.6 | 0.003 | 121.5 |
| 36 | G4 | *y = β_0_ + β_1_ LV + β_2_ BPA* | -10 | 9.7 | 0.003 | 126.3 |
| 37 | G4 | *y = β_0_ + β_1_ LV + β_2_ PPA + β_3_ BLC* | -9.1 | 10.6 | 0.002 | 199.1 |
| 38 | G2 | *y = β_0_ + β_1_ PPA + β_2_ PLC + β_3_ PLD* | -8.9 | 10.7 | 0.002 | 213.6 |
| 39 | G4 | *y = β_0_ + β_1_ LV + β_2_ PLC + β_3_ BPA* | -8.8 | 10.9 | 0.002 | 233.4 |
| 40 | G4 | *y = β_0_ + β_1_ LV + β_2_ PPA + β_3_ BPA* | -8.3 | 11.3 | 0.001 | 288.9 |

AICcΔ - differences in AICc relative to the lowest value of AICc of all models; AICcW_i_ - Akaike weight of model i; W_1_ / W_i_ - ratio between the weight of model 1 and the weight of the respective model; G1 - Local vegetation; G2 - Proximal landscape structure; G3 - Broad landscape structure; G4 Multi-level combined effect; Null model – no effect; *β_0_* - intercept; *β_1_*, *β_2_* and *β_3_* - parameters associated with the respective variables; *LV* - local vegetation; *PPA* – Proximal landscape proportion of agricultural cover; *PLC* - Proximal landscape configuration; *PLD* - Proximal landscape diversity; *BPA* – Broad landscape proportion of agricultural cover; *BLC* - Broad landscape configuration; *BLD* - Broad landscape diversity.
